# The dimensionality of plant–plant competition

**DOI:** 10.1038/s41467-026-76980-9

**Published:** 2026-08-26

**Authors:** Daniel B. Stouffer, Oscar Godoy, Giulio V. Dalla Riva, Margaret M. Mayfield

**Affiliations:** 1 Department of Evolutionary and Integrative Ecology, Leibniz Institute of Freshwater Ecology and Inland Fisheries (IGB), Berlin, Germany; 2 School of Biological Sciences, University of Canterbury, Christchurch, New Zealand; 3 Estación Biológica de Doñana (EBD-CSIC), Sevilla, Spain; 4 Department of Mathematics & Statistics, University of Canterbury, Christchurch, New Zealand; 5 School of Biological Sciences, University of Queensland, Brisbane, QLD Australia; 6 School of BioSciences, The University of Melbourne, Parkville, VIC Australia

**Keywords:** Community ecology, Ecological modelling, Biodiversity, Theoretical ecology

## Abstract

To avoid extinction, every species must be able to exploit available resources at least as well as all other species in its community. Theory predicts that, the more distinct the niches of competing species, the more species that can coexist together. However, both theoretical and experimental studies define a priori the nature and number of resources over which species presumably compete. It therefore remains unclear whether species in empirically realistic contexts actually fill all of the niches available to them. Here, we show how the interactions between co-occurring species can be used to quantify their implied “niche dimensionality”: the effective number of axes over which those species show niche differentiation. We then apply this approach to data from 12 plant assemblages distributed across the globe. Contrary to expectation, we find that their niche dimensionality was much lower than the number of species. However, data from two high-resolution experiments show that changes to the local environment reshuffle plants’ competitive roles and hence act to increase the assemblages’ emergent niche dimensionality. Our results therefore reinforce the notion that homogeneous environments are less capable of maintaining high diversity while also highlighting how environmental variation shapes species’ niches and hence moderates their long-term survival.

## Abstract (web summary)

Theory posits that competing species should occupy different niches to coexist, and this should be detectable in the interactions between them. In contrast, a new method indicates that plant-plant interactions show very limited niche differentiation.

## Introduction

Much of our current understanding of species coexistence derives from studying exploitation competition—competition between similar species for a shared pool of finite, limiting resources such as water, nutrients, light, or space^1–6^. In relatively constant environments, the coexistence of many species is strongly influenced by two conditions above all others: there should be at least as many resources available as coexisting species^7–9^ and those species should have different niches (e.g., resource-use requirements)^10–13^. When species have distinct niches—for example, by specializing on unique resources^14,15^—intraspecific competition should exceed interspecific competition, and this can in turn help prevent communities from becoming overrun by the most dominant competitor or competitors^16,17^. When species are functionally similar, in contrast, their coexistence may benefit from the presence of additional, external sources of variability to buffer against otherwise unfavorable years, habitats, or environmental conditions^18,19^. Indeed, ecological drift alone can be sufficient to exclude all but one species from any finite community whose constituent species show no niche differentiation^20,21^.

The theoretical basis linking niche differentiation to coexistence is quite clear^22–25^. The importance of niche differences for maintaining species diversity also has strong empirical support^26–28^. The link between theory and empirical observations is, however, still unclear since there is no clear picture of the number of niche dimensions that are actually exploited within diverse natural communities. This is partly due to the fact that researchers traditionally select a priori the relevant resources or limiting factors over which species are thought to compete^8,29–31^. Unfortunately, such approaches cannot guarantee that all relevant dimensions have been taken into account, or that those selected are even the most relevant ones in practice; even controlled scenarios can overlook previously-unknown, but equally-important, mechanisms. To manage these complications, some researchers have taken a more indirect approach of relating the strength of competition and resource-use variation to differences in functional traits^13^ or evolutionary histories^32,33^, based on the assumption that these species characteristics are reasonable summaries of the multiple dimensions that compose a species’ niche. Yet indirect, correlative approaches also cannot conclusively identify which resources are limiting^8^ or, more importantly, how many niche dimensions are realized^34^. It therefore remains unclear whether or not the interactions between species in any given ecological assemblage are indicative of highly differentiated or highly similar species competing over a large or a small resource pool (Supplementary Note S1).

As tantalizing as it might be, exhaustive inference of niche dimensionality in diverse empirical communities—as achieved by direct manipulation of all potential niche axes (e.g., light, water, space, nutrients, pollinators, natural enemies, etc.)—is next to impossible. With this in mind, we introduce here an alternative perspective on this long-standing question. Rather than characterize and compare resource use explicitly or select proxies from species’ characteristics, we develop an approach that uses information about the strengths and signs of interactions between species to infer their implied niche basis (see “Mathematical framework for estimating niche dimensionality”). In particular, our method provides a means to quantify the “niche dimensionality” of any interacting species assemblage. Conceptually, niche dimensionality can be thought of as a proxy for the number of limiting factors over which those species show niche variation (Supplementary Note S2). In addition, our framework implies that it is also a data-informed and mathematical property of the emergent interactions between species.

In addition to estimating niche dimensionality, our approach decomposes pairwise interactions between species into (i) species’ response traits, (ii) species’ effect traits, and (iii) the relative strength of these responses and effects across the underlying niche dimensions (see “Mathematical framework for estimating niche dimensionality”). This decomposition helps us identify species’ competitive strategies across the niche dimensions shared among the assemblage (Supplementary Note S2). Specifically, effect traits modulate the impact of each species on all others while response traits modulate the impact of all other species on each species^17,35^. When combined, the effect traits of one species and the response traits of another determine whether their directed, pairwise interactions will be strong or weak, competitive or facilitative^32,36–38^. Given that the net strength of an interaction between two species should be proportional to their niche overlap^7,39^, our framework quantifies species’ niche differences in terms of the extent to which they have an ability to withstand neighbour effects (their response) as well as an ability to generate neighbour effects (their effect). Along each niche dimension, each set of response and effect traits thus relates to a comparable (albeit latent and unobservable) set of “effective resources” such that a species with a large effect trait can be thought of as one that depletes the corresponding resource to the detriment of others, and a species with a large response trait can be thought of as one that is particularly sensitive to scarcity of that same resource. As such, response and effect traits allow us to capture the multi-dimensional strategies employed by species to outcompete each other or to avoid being outcompeted^34,35^ based directly on the observed outcomes of species–species interactions (as opposed to indirectly; e.g., via their phenotypic traits^40^).

In this work, we apply our approach to data from 12 empirical assemblages drawn from plant communities across the globe (see “Methods”). These assemblages cover a broad array of habitat types and plant life-history strategies, from deserts to forests and annual plants to trees (Table 1). Each dataset includes information regarding the strength and sign of species interactions between three to ten different plant species, and allows us to determine the extent to which each species performs better or worse in the presence of all others. Moreover, the data come from field or common garden settings—in which we might expect extrinsic factors could give rise to greater realized variation—and greenhouses—in which we might expect reduced environmental heterogeneity and hence realized variation should be driven almost entirely by intrinsic species properties. Studying these data allows us to investigate whether there are common patterns of niche dimensionality across a broad diversity of ecological communities. In general, we observe that niche dimensionality of each assemblage is much lower than the number of species therein. We also find that changing environmental conditions act to increase an assemblage’s effective niche dimensionality, and using a toy model, we illustrate how this increase can lead to improved prospects of coexistence. Overall, our study challenges a key assumption regarding the structure underpinning interactions in diverse communities while also demonstrating the importance of the abiotic environment in shaping species’ realized niches.

**Table 1:** Dataset details and inferred niche dimensionality

Dataset	Country	Habitat	Experimental setting	Experimental setting	Experimental setting	Species richness (*S*)	Niche dimensionality ($\hat{d}$)	Reference
1	ESP	Grassland	Field	Wet	Raw	10	2	Matías et al. ^51^
1	ESP	Grassland	Field	Dry	Raw	10	3	Matías et al. ^51^
2	AUS	Open woodland	Field	Sun	Raw	8	1	Stouffer et al.^63^
2	AUS	Open woodland	Field	Shade	Raw	8	2	Stouffer et al.^63^
3	USA	Old field	Greenhouse	–	Matrix	7	1	Goldberg & Landa^35^
4	USA	Shrubland	Garden	–	Matrix	7	2	Kinlock^41^
5	PAN	Tropical forest	Field	–	Matrix	5	3	Svenning et al.^80^
6	CHN	Desert	Greenhouse	–	Matrix	4	2	Gao et al.^81^
7	BRA	Estuarine	Field	–	Matrix	3	2	Costa et al.^82^
8	ESP	Old field	Garden	–	Matrix	3	2	Doménech & Vilá^83^
9	USA	Grassland	Field	–	Matrix	3	2	Farrer & Goldberg^84^
10	USA	Old field	Greenhouse	–	Matrix	3	1	Gurevitch et al.^85^
11	USA	Grassland	Greenhouse	–	Matrix	3	1	Hedberg et al.^86^
12	GER	Grassland	Garden	–	Matrix	3	1	Weigelt et al.^87^

For each plant assemblage, we show its Country of origin, the Habitat the species are drawn from, the Experimental setting, the corresponding Environmental treatment (if applicable), whether we have empirical data with which to fit a model for density-dependent performance (Raw) or a previously inferred matrix of interaction coefficients (Matrix), the Species richness *S*, the inferred Niche dimensionality $\hat{d}$, and the original Reference within which to find all experimental protocols.

## Results

### Mathematical framework for estimating niche dimensionality

For each assemblage of *S* plant species, at the core of our approach sits an *S* × *S* pairwise interaction matrix **A**. In this interaction matrix, the element *α*_*ij*_ in row *i* and column *j* gives the per capita strength of the effect of species *j* on species *i*. Rather than assume that each of these values is unique, we instead aim to provide as accurate an approximation of them as possible^32,38^. To do so, our mathematical framework provides a principled way to split every pairwise interaction *α*_*ij*_ into *d* separate components, which we refer to as “niche dimensions”.

For any integer value of 1 ≤ *d* ≤ *S*, we define the per capita strength of the effect of species *j* on species *i* as

1 $${\alpha }_{{ij}\big| d }={\sum }_{k=1}^{d}{\sigma }_{k}\,{r}_{i,k}\,{e}_{j,k}\,.$$

The sum here is across the *d* different niche dimensions, and each of these is weighted by the dimension’s average strength of interactions (*σ*_*k*_ > 0). For the simplest case of *d* = 1, the per capita strength of the effect of any species *j* on any species *i* is given by *α*_*ij*∣*d* = 1_ = *σ*_1_ *r*_*i*,1_ *e*_*j*,1_; that is, the product of the average strength of interactions in the first dimension (*σ*_1_), the “response trait” of species *i* in the first dimension (*r*_*i*,1_), and the “effect trait” of species *j* in the first dimension (*e*_*j*,1_). We refer to the *r*_*i*,*k*_ parameters as response traits because they influence how the performance of the same focal species *i* “responds” to the presence of different neighbour plants; similarly, we refer to the *e*_*j*,*k*_ parameters as effect traits because they influence how the same neighbour plant “effects” the performances of different focal plants. Importantly, the separation of responses from effects allows us to generate asymmetric interaction matrices—where the effect of species *i* on *j* differs from the effect of species *j* on *i*—which is consistent with patterns observed in empirical data^38,41,42^.

### Estimating best-fit reduced-dimensionality model parameters

When we had data consisting of a previously inferred empirical interaction matrix **A** for a given assemblage, we determined the maximum-likelihood parameters for Eq. (1) using singular value decomposition (*SVD*)^43^. Specifically, *SVD* can be used to factorize **A** into three separate matrices such that **A** ≡ **R** **Σ** **E**^*T*^. Here, **R** and **E** are *S* × *S* orthogonal matrices (i.e., matrices whose columns and rows are all orthogonal unit vectors), and **Σ** is an *S* × *S* matrix with the decreasing singular values of **A** along the diagonal (i.e., *σ*_1_ > *σ*_2_ > ⋯ > *σ*_*S*_ ≥ 0) and zeroes elsewhere. We show in the Supplementary Note S3 why *SVD* is mathematically equivalent to the additive partition scheme provided by Eq. (1). At every niche dimensionality 1 ≤ *d* < *S*, *SVD* works in such a way that ${\hat{{{{\bf{A}}}}}}_{d}={\hat{{{{\bf{R}}}}}}_{d}\,{\hat{{{{\mathbf{\Sigma }}}}}}_{d}\,{\hat{{{{\bf{E}}}}}}_{d}^{T}$ is the best least-squares approximation of **A** when: ${\hat{{{{\bf{R}}}}}}_{d}$ is the *S* × *d* sub-matrix given by the first *d* columns of *R*, ${\hat{{{{\bf{E}}}}}}_{d}$ is the *S* × *d* sub-matrix given by the first *d* columns of *E*, and ${\hat{{{{\mathbf{\Sigma }}}}}}_{d}$ contains the *d* largest singular values of **A**.

When we had raw empirical data for which the values composing the interaction matrix **A** were a priori unknown, we inferred them by fitting a model of density-dependent plant performance to the empirical data (see “Methods”). For any niche dimensionality 1 ≤ *d* < *S*, this required estimating the *d* positively constrained values along the diagonal of ${\hat{{{{\mathbf{\Sigma }}}}}}_{d}$ and the values within the two *S* × *d* matrices ${\hat{{{{\bf{R}}}}}}_{d}$ and ${\hat{{{{\bf{E}}}}}}_{d}$. To avoid over-parameterization (and to maintain coherence with *SVD*), we constrained the columns of both ${\hat{{{{\bf{R}}}}}}_{d}$ and ${\hat{{{{\bf{E}}}}}}_{d}$ to be orthogonal unit vectors. For each of these two matrices, this implies that their first column has *S*−1 free parameters, their second column has *S*−2 free parameters, and so on, up to a maximum of $S\left(S-1\right)/2$ free parameters per matrix when *d* = *S*. In total, we must therefore perform inference on $d\left(2S-d\right)$ parameters. Though our specific orthogonality constraints are not the only possible solution to this problem, it is important to note that it will always be necessary to impose constraints on the matrices ${\hat{{{{\bf{R}}}}}}_{d}$, ${\hat{{{{\bf{E}}}}}}_{d}$, and ${\hat{{{{\mathbf{\Sigma }}}}}}_{d}$ to avoid issues of unidentifiability. This is because the matrix product ${\hat{{{{\bf{R}}}}}}_{d}\,{\hat{{{{\mathbf{\Sigma }}}}}}_{d}\,{\hat{{{{\bf{E}}}}}}_{d}^{T}$ will always produce a rank-*d* matrix ${\hat{{{{\bf{A}}}}}}_{d}$ of size *S* × *S* which has exactly $d\left(2S-d\right)$ degrees of freedom.

There are at least three salient points regarding our inference process that are worth highlighting explicitly. First, even if species interactions were the result of real-world response and effect traits that were not orthonormal, our inference process built around *SVD* will always return estimated ${\hat{{{{\bf{R}}}}}}_{d}$ and ${\hat{{{{\bf{E}}}}}}_{d}$ that are. Otherwise, we risk over-parameterizing the problem and suffer from the aforementioned identifiability problems^44,45^. This is also why we noted above that the dimensions of those matrices relate to latent, “effective resources” (see also Supplementary Notes S1 and S2). Second (and building from the first point), the orthogonal nature of ${\hat{{{{\bf{R}}}}}}_{d}$ and ${\hat{{{{\bf{E}}}}}}_{d}$ implies that they should be thought of as providing detail about where the different species sit relative to each other in the assemblage’s overall competitive hierarchy. Third (and building from the second point), ${\hat{{{{\bf{R}}}}}}_{d}$ and ${\hat{{{{\bf{E}}}}}}_{d}$ are best thought of as plastic species traits that are indicative of the conditions under which the interactions took place, both biotic (e.g., the set of species that were interacting) and abiotic (e.g., the types of resources that are available). Consequently, realized niche dimensionality of an assemblage will always reflect both the fundamental niche variation between species and which resources are locally most limiting (Supplementary Notes S1 and S2).

### Niche dimensionality and species coexistence

Mathematically, an *S* × *S* interaction matrix **A**_*d*_ that is exactly rank-*d* is “rank deficient”. This means that, when embedded in typical population-dynamics models, the matrix **A**_*d*_ imposes a strict limit such that *d* is the maximum number of species that could coexist in the absence of exogenous variation^7,9,10,46^. We can therefore get an estimate of the ease with which species from an empirical assemblage can achieve multi-species coexistence by examining the extent to which the *d*-dimensional approximation **A**_*d*_ captures the variation in their un-approximated interaction matrix **A**. As we scan across the values ${\hat{\sigma }}_{1}$, ${\hat{\sigma }}_{2}$, …, ${\hat{\sigma }}_{S}$, we expect their magnitudes to decrease much more quickly at first before leveling off close to zero^47,48^. For each assemblage, there should therefore be a point $\hat{d}$ beyond which the inclusion of additional dimensions will only weakly change the interaction strengths. We refer to this value $\hat{d}$ as the assemblage’s “niche dimensionality”, where $\hat{d}$ is number of niche dimensions required to explain at least 95% of the variation observed within the data. Though all values $d > \hat{d}$ will give a more exact description of species’ fundamental niches, we argue that $\hat{d}$ is a better reflection of the niche dimensions that underpin the realized species–species interactions. Even when the matrix **A** has not been approximated (i.e., there is no strict mathematical limit to the maximum number of species that could potentially coexist), the value of $\hat{d}$ inferred from that matrix implies that it should be possible for at least some *n*-species subsets from the assemblage to coexist (when $n\le \hat{d}$ and species’ fitness differences allow it^23^) but will be increasingly challenging for all *n*-species subsets comprising more than $\hat{d}$ species.

### Inferred niche dimensionality of empirical datasets

**Fig. 1:**
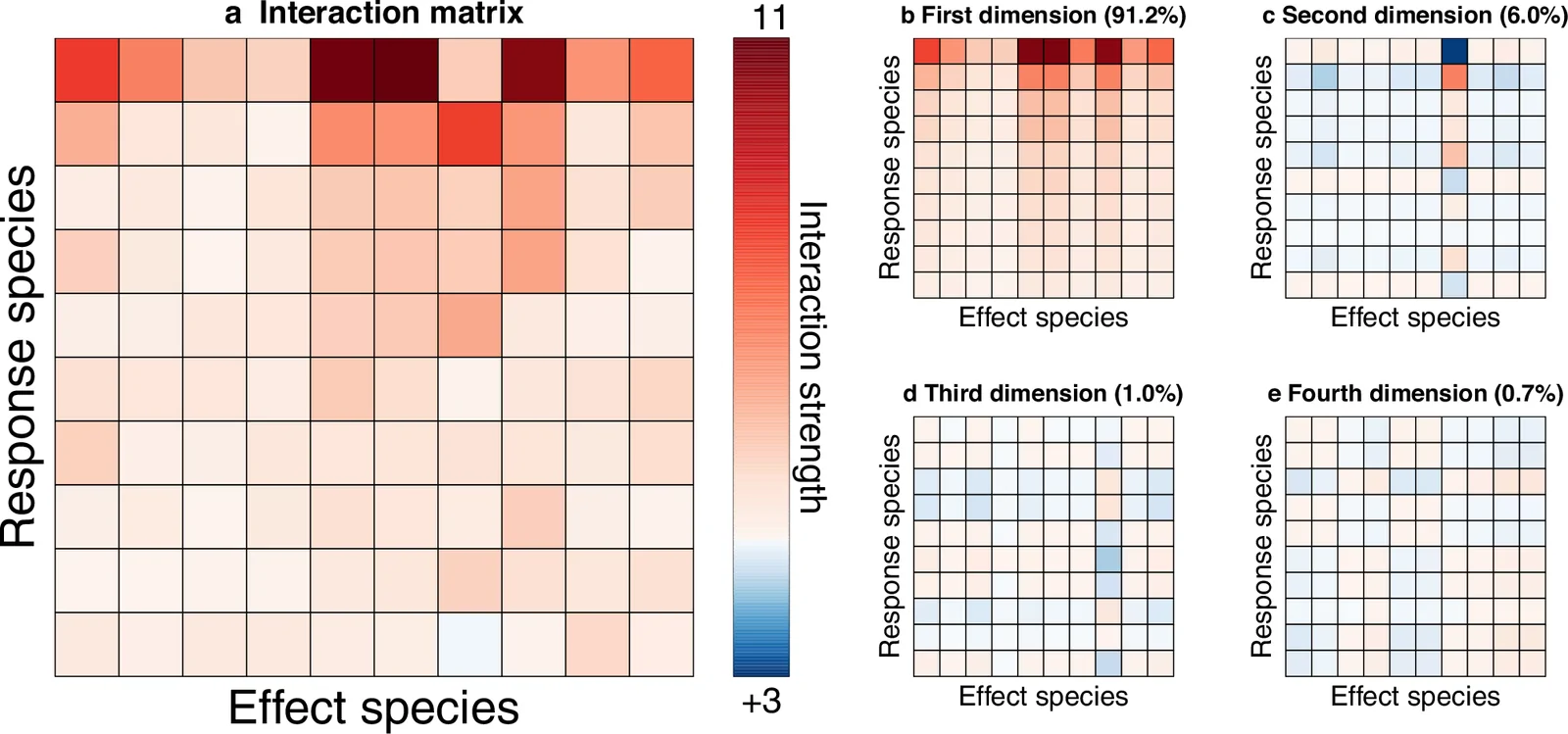
Response-effect decomposition of a pairwise interaction matrix. **a** The inferred 10 × 10 interaction matrix for Dataset 1-Wet, where rows indicate the species responding to the interaction and the columns represent the species effecting the interaction. Interactions are colored based on the strength and sign of the net pairwise impact: net competitive values are indicated from white to red, while net facilitative values are indicated from white to blue. The full pairwise interaction matrix can be parsimoniously decomposed into 10 matrices that capture implicit niche differentiation underpinning interactions across the assemblage (see “Mathematical framework for estimating niche dimensionality” & Supplementary Note S1). **b**–**e** show the four leading dimensions of such a decomposition following Eq. (1). For this dataset, the first, second, third, and fourth niche dimensions explain 91.2, 6.0, 1.0, and 0.7% of the variation observed in the data, respectively. Note that interactions in the first niche dimension are overwhelmingly competitive whereas species-specific niche variation within the second through fourth dimensions both strengthen and weaken the net strength of pairwise interactions.

We started by determining the niche dimensionality $\hat{d}$ of all 12 plant assemblages in our data (see “Methods”). As noted above, niche dimensionality $\hat{d}$ gives an indication of the effective number of resources over which the species are likely competing. For every dataset and regardless of the experimental context, we observed that three or fewer niche dimensions ($\hat{d}\le 3$) were sufficient to accurately capture the pairwise interactions between plant species (Table 1). In fact, the first niche dimension alone explained on average 86.7% of the variation in the plant–plant interactions, and this ranged from 59.6 to 99.3% across the 12 datasets (Fig. 1 and Supplementary Notes S4 and S5). To verify that these low values of $\hat{d}$ are an ecological feature of these species assemblages and not merely an artefact of our methodology, we also estimated the niche dimensionality for simulated data with randomly-assigned interaction coefficients and within randomizations of the empirical interaction matrices. As expected, randomization tended to create unstructured data with niche dimensionality greater than observed in the natural and experimental assemblages (Supplementary Note S6).

**Fig. 2:**
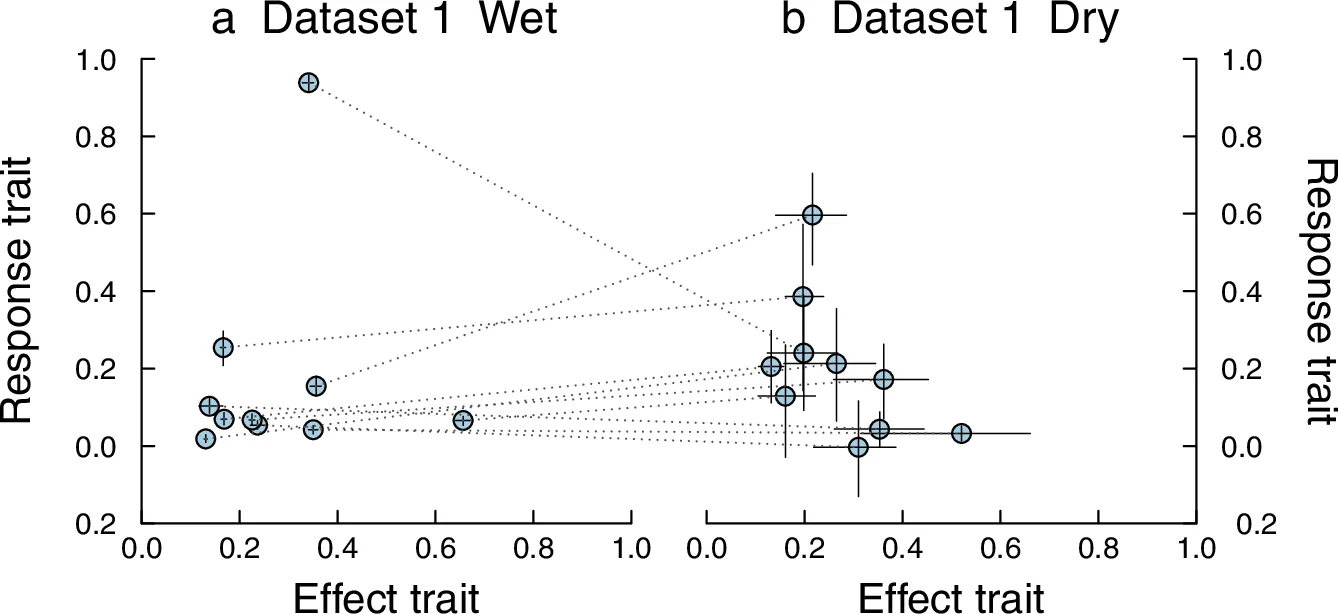
Species’ response and effect traits in the first niche dimension for both Dataset 1-Wet and Dataset 1-Dry. **a** In Dataset 1-Wet, species are organized along a competitive hierarchy driven largely by variation in their effects as neighbours: dominant competitors have large positive effect traits and small positive response traits, whereas weaker competitors have small positive effect traits and large positive response traits. One notable species clusters outside this hierarchy by exhibiting a moderate effect trait and large positive response trait, indicating that it is particularly susceptible to competitive effects. **b** In Dataset 2-Dry, species are again organized in a competitive hierarchy but exhibiting clearer variation in both response and effect traits. The dotted lines connect species' response and effect traits in Wet environmental conditions to those same species' response and effect traits in Dry environmental conditions. Variation between where species fall in the two panels is thus indicative of reorganization of the underlying competitive hierarchy. In both panels, the points indicate the median inferred value and the error bars at each point indicate the 25th to 75th percentile confidence interval; the error bars have been plotted on top of points to facilitative visibility when they are small.

We next examined species’ inferred response and effect traits to get an idea about the forces underpinning species interactions. As representative examples, the best-fit parameters for Dataset 1-Wet and Dataset 1-Dry indicate that the 10 constituent species play distinct ecological roles across their leading niche dimension (Fig. 2). In both cases, species appear to be sorted into classic competitive hierarchies within which species with strong effects tended to have weak responses and those with weak effects tended to have strong responses. However, realized niche differentiation in Dataset 1-Wet was driven by greater variation in species’ effects than their responses, whereas species had heterogeneous responses and heterogeneous effects in Dataset 1-Dry. We observed similar patterns across each of the empirical datasets (Supplementary Note S7), indicating that the leading dimensions of niche differentiation are those that create variation in species’ responses to and effects on other species in their community.

**Fig. 3:**
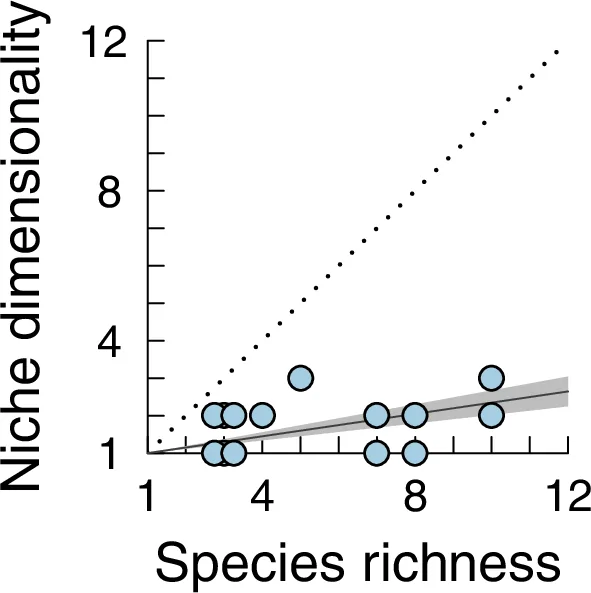
Inferred niche dimensionality ($\hat{d}$) as a function of the species richness of each empirical assemblage (*S*). The dotted line represents the upper bound of niche dimensionality equal to the number of species. The solid line represents the predicted increase of niche dimensions ( ± standard error) based on a linear regression through the observed species richness and inferred niche dimensionality values across the empirical datasets. Note that this trend line has been constrained to pass through $\hat{d}=1$ when *S* = 1 since a one species assemblage necessarily requires only a single niche dimension. Datasets with identical values of $\hat{d}$ and *S* have been jittered slightly along the *x*-axis to make them visible.

We consistently found that estimates of niche dimensionality $\hat{d}$ were lower than the total number of species *S* in each empirical assemblage. Furthermore, this did not depend on whether or not data came from experiments in the field, garden, or greenhouse (Table 1). Though our estimates of niche dimensionality increased with increasing species richness (Fig. 3), the rate of increase was much lower than one dimension per species as expected from theory^7–9,11^ and lower than what we observed in randomizations of the empirical data (Supplementary Note S6). When niche dimensionality is less than the number of species in an assemblage, the interactions of two or more species directly mirror each other^10^. Furthermore, should any among a set of functionally-similar species have even the slightest competitive advantage, it will tend to dominate the others in that set over the long run^20^. Our results, therefore, are indicative of communities for which many-species coexistence should pose a serious challenge. Though this observation runs contrary to high local diversities found in nature, it may provide an explanation for why numerous previous studies have found that models parameterized with empirical data very rarely predict coexistence^13,49,50^.

### Inferred niche dimensionality under different environmental conditions

To investigate this idea more deeply, we next determined whether changing environmental conditions impact plant species’ roles within their assemblage’s competitive hierarchy. To do so, we explored two of our datasets in greater detail as they each comprise the same underlying species assemblages, but with interactions between those species taking place in two distinct environments. Specifically, one pair of datasets (Dataset 1-Wet & Dataset 1-Dry) consists of ten species in control and simulated-drought conditions^51^, designed to mimic the impact of extreme climatic events of the sort expected with ongoing global change^52^; the second pair of datasets (Dataset 2-Sun & Dataset 2-Shade) consists of eight species in control and artificially-shaded conditions, since light availability is known to structure local diversity in that system^53^. Viewed independently, Dataset 1 was captured by two and three niche dimensions in the Wet and Dry environments, respectively; Dataset 2 was captured by one and two niche dimensions in the Sun and Shade environments, respectively. Nevertheless, the question remains whether the species in each Dataset sorted themselves along the same leading axes of niche variation in the contrasting environments. To check this, we measured the correlation between species’ inferred response and effect traits as realized under the two environmental conditions (see “Methods”). These correlations were weak and non-significant (Procrustes *ρ* = 0.52, *p* = 0.37 for Dataset 1 when compared to *n* = 999 random permutations; Procrustes *ρ* = 0.40, *p* = 0.06 for Dataset 2 when compared to *n* = 999 random permutations), indicating that the niche dimensions being exploited by both sets of species likely differ following changes in environmental conditions. Environmental variation, therefore, leads to significant shifts in species’ niches (Figs. 2 and S7), in contrast to niche theories based on environmental tolerance^54^ or functional traits^55^, which generally assume that niches are immutable species’ attributes.

### Low-dimensional coexistence is achievable in varying environments

Motivated in particular by our results for Dataset 1, we lastly put together a toy model to explore whether environmentally driven variation of species’ response and effect traits can potentially increase the likelihood of coexistence (Supplementary Note S8). Starting from a widely studied model for competing annual-plant species^56,57^, we parameterized the model for two species (*i* and *j*) such that species *i* is favoured in Wet conditions and species *j* is favoured in Dry conditions. Within our response–effect parameterization, this was achieved by species *i* having a weaker response to the presence of neighbours in Wet conditions than did species *j*, and the converse in Dry conditions. In all other facets of the model, the two species are ecologically equivalent^58^. As a result, the species’ dominance hierarchy flips when shifting between the two types of environments.

Because we constrained the interactions between these two species in our toy model to occur at niche dimensionality *d* = 1, it is mathematically impossible for the two species to coexist in the long term under fixed environmental conditions because they lack niche differences^23,46^, and simulations of the model in perpetually Wet or Dry environments bear out this result (Fig. S15). Indeed, in either constant environment no amount of fitness differences will ever be able to overcome the barrier presented by a lack of niche differences between these two species^20^. If, on the other hand, those same species compete in an environment that changes from Wet to Dry conditions (and back) over time, both species may be able to avoid competitive exclusion in the long run (Fig. S15). Since each species remains vulnerable to prolonged exposure to its less preferred environment, environmental variation is not a sufficient condition for long-term coexistence^17^. Nevertheless, environmentally driven changes in species’ interaction patterns—as modulated by variation in their response and/or effect traits changing from one set of conditions to another—can provide a plausible means through which they may maintain a firmer foothold.

## Discussion

We provide strong evidence that plant–plant interactions are organized over a small number of niche dimensions. Intriguingly, this observation holds despite the differences between the plant communities represented in our study—such as their biogeographical provenance, evolutionary histories, or plant growth form. Less clear is whether the emergent pattern is more a consequence of the plant assemblages exhibiting a striking lack of fundamental niche differences or competing over a small number of truly limiting resources. Our exploration of a common consumer–resource model emphasizes that the number of distinct features limiting plant populations, such as the number of limiting resources or species’ degrees of resource specialization, can differ substantially from the number of dimensions realized in their interaction matrix (Supplementary Note S2). To convincingly tease these two apart in the future, we suspect ecologists may require a clever combination of manipulative and observational experiments beyond those that exclusively serve to estimate one set of interaction strengths. These could include invasibility experiments conducted in different environments^17^ or manipulating as many potential limiting factors as possible and seeing how interaction strengths—even for only a subset of species of interest—respond as a result^59^.

Regardless of its root cause, low niche dimensionality would appear to imply limited prospects for many-species coexistence and, notably, belies common theoretical expectations. This notwithstanding, limited variation in terms of species’ interactive responses and effects is actually in strong agreement with other assessments of global plant diversity being captured by a small number of life histories^34^ and limited phenotypic trait combinations^55,60^. Though disentangling the underlying drivers of species’ response and effect traits inferred across our empirical datasets was beyond the scope of this particular study, at least one immediate extension would be to explore whether or not these latent traits can be related to measurable phenotypic characters across the species in question, such as root or leaf morphology.

In addition to demonstrating that low-dimensional competition is pervasive in plant assemblages, our approach provides a new lens through which to interrogate coexistence in varying environments. Previous studies of the species found in Datasets 1 & 2 also predicted that the number of species expected to coexist in a single environment is generally small^51,61,62^; those studies’ predictions closely align with our own estimates of both assemblages’ niche dimensionality. Likewise, results from those same two systems found that which species are expected to coexist changes from one set of environmental conditions to another^51,61–63^. If we combine these previous observations with our own results, it appears that the environment can leave such a strong imprint on interactions that its variability behaves conceptually like an additional niche dimension and thus provides a de facto landscape across which species can evolve novel strategies to avoid being outcompeted^27,34,64^. While this can make it harder to identify consistent structural patterns in empirical interaction matrices^41,42,65^, it also highlights a critical link between changing environmental conditions and species’ realized niches^64,66^.

Much research is dedicated to exploring how the abiotic environment impacts species’ performance in an interaction-free context^67,68^. Indeed, most predictions about how communities will change in the face of external disturbance, such as climate variability, hinge on this relationship^52,54^. Comparatively less is known about how environmental conditions impact pairwise interactions in multi-species assemblages and therefore about species’ ability to persist under changing environmental conditions^42,51,69–72^. One possible reason is that it is difficult to relate changes across multivariate interactions between species to changes in univariate single-species outcomes^25,50^. Here, we show how to decompose pairwise interactions into species-level response and effect traits, thereby providing a potential simplification for such inquiries.

Moreover, the impact of the environment on species’ response and effect traits generates multiple ecologically-relevant ways for pairwise interactions to vary along environmental gradients. All else being equal, any species whose response traits decrease in value from one environment to another will suffer less from competition; in contrast, any species whose effect traits increase in value from one environment to another should become increasingly dominant. Both pathways can assist in species avoiding being competitively excluded. Going forward, we therefore expect it will be particularly exciting to determine which one of these tendencies predominates in nature. Of particular utility here would be more studies like Datasets 1 & 2, but with interactions measured across multiple points along continuous, as opposed to categorical, environmental gradients.

## Methods

### Empirical data

We analyzed data from 12 empirical assemblages to test our core hypothesis regarding variation in the structure of pairwise interactions between co-occurring plants (Table 1). For Datasets 1-Wet, 1-Dry, 2-Sun, and 2-Shade, we directly analyzed raw empirical data from experimental studies of competition between annual plants in order to statistically infer the interaction strengths that make up the pairwise interaction matrix **A**. For Datasets 3–12, we analyzed previously-estimated pairwise interaction matrices **A** that were available in the literature. All such interactions were inferred based on the performance of plant individuals growing in isolation relative to individuals growing in the presence of conspecific and/or heterospecific neighbours^41^.

### Inferring fixed-dimensionality interaction strengths from empirical data

Each of Datasets 1-Wet, 1-Dry, 2-Sun, and 2-Shade consists of estimates of individual plant performance and the abundance or density of co-occurring plants within interaction neighbourhoods^51,63^. To infer interaction strengths that make up their corresponding matrix **A**, we therefore first had to define a mathematical model for how performance varies as a function of neighbour composition and abundance. In line with current best practice^37^, we estimated the per capita effects of neighbouring species on the performance of focal individuals of each of the datasets using a model of the form

2 $${F}_{i}=\frac{{\lambda }_{i}}{1+\mathop{\sum }_{j=1}^{S}{\alpha }_{ij| d}{N}_{j}}\,,$$

where *F*_*i*_ is the observed estimate of the performance of a focal individual from species *i*, *λ*_*i*_ captures intrinsic performance of these individuals in the absence of competition, *α*_*ij*∣*d*_ is the per capita impact of species *j* on species *i*, *N*_*j*_ is the abundance of species *j* in the focal individual’s interaction neighbourhood, and the sum is across all species in that neighbourhood (which could potentially include conspecifics of species *i*). Given data of focal-plant performance and those plants’ interaction neighbourhoods, inference of both intrinsic performance and the strength of pairwise interactions can usually be achieved with standard regression approaches^73^. For example, Eq. (2) with count responses (as in Dataset 1 and Dataset 2), can be fit as a Poisson regression with inverse link function^74^. However, these regression approaches are no longer feasible when requiring interactions to occur at a fixed niche dimensionality due to our orthgonality constraints. We therefore used the *mle2* function from the *bbmle* package^75^ in the statistical programming language R^76^ to identify the best-fit $d\left(2S-d\right)$ total parameters that make up the optimal values of ${\hat{{{{\mathbf{\Sigma }}}}}}_{d}$, ${\hat{{{{\bf{R}}}}}}_{d}$, and ${\hat{{{{\bf{E}}}}}}_{d}$ at each niche dimensionality 1 ≤ *d* < *S*. In order to ensure that the parameters of ${\hat{{{{\bf{R}}}}}}_{d}$ and ${\hat{{{{\bf{E}}}}}}_{d}$ maintain our orthogonality constraints and uniqueness during optimization, we followed the Rational Cayley Transform method of refs. ^44,45^. To facilitate comparison to the approach with which the matrix **A** is composed entirely of free parameters (i.e., when *d* = *S*), we used *mle2* to minimize the exact same deviance function as used for Poisson regression rather than to maximize the data’s log-likelihood.

### Statistical analysis

For Datasets 1-Wet, 1-Dry, 2-Sun, and 2-Shade, we inferred the best-fit parameters for interactions constrained to occur when dimensionality *d* = *S*. We then used the inferred values of $\{{\hat{\sigma }}_{1},{\hat{\sigma }}_{2},\ldots,{\hat{\sigma }}_{S}\}$ and identified the niche dimensionality $\hat{d}$ that was sufficient to capture 95% of the variation in the inferred interaction matrix. For Datasets 3–12—for which we only had an estimated interaction matrix **A**—we determined the niche dimensionality $\hat{d}$ based directly on the singular values of **A** with an equivalent threshold of 95%. In both instances, the variance explained by each dimension *j* can be calculated as ${\sigma }_{j}^{2}/\mathop{\sum }_{k=1}^{d}{\sigma }_{k}^{2}$. For all dataset types, values $d < \hat{d}$ fail to capture biologically meaningful variation in the observed plant–plant interactions; values of $d > \hat{d}$ require the use of an over-parameterized statistical model with an excess of resource dimensions. We note that our 95% threshold may be a conservative test of our theory. However, to the best of our knowledge, there is no other rule of thumb with which to decide precisely how much variation is biologically meaningful.

For the paired Datasets 1 & 2, we tested whether the positions of species in their underlying $\hat{d}$-dimensional trait spaces were correlated using a Procrustes analysis. Since the inferred niche dimensionalities differed across environmental conditions (Table 1), we first converted the two $S\times \hat{d}$ matrices ${\hat{{{{\bf{R}}}}}}_{d}$ and ${\hat{{{{\bf{E}}}}}}_{d}$ (for each environmental condition in each Dataset) into a single *S* × *S* distance matrix whose elements were the Euclidean distances between species’ vectors of response and effect traits. We then used the Procrustes analysis to estimate the similarity between the distance matrices computed using the best-fit parameters in each environmental condition^77^. A significant Procrustes statistic indicates that the effective response and effect hierarchies between species in both environments are positively related (e.g., the same species always tend to be the strongest or weakest effectors or responders), whereas a non-significant result implies that species likely play different roles when the environmental conditions change. We used the *protest* function from the *vegan* package^78^ in the statistical programming language R^76^ to perform our Procrustes tests.

## Supplementary information

Supplementary Information

Transparent Peer Review file

## Data Availability

All data used here were drawn from previously published studies and can be obtained from publicly available repositories. Dataset 1 can be obtained from 10.5061/dryad.5d1s9. Dataset 2 can be obtained from 10.5061/dryad.8v13t2q. Datasets 3–12 form part of data available at 10.5061/dryad.1sm06sp. More details regarding the original studies can be found in the references as indicated in Table 1.

## Code availability

The data and code necessary to recreate the analyses presented in this manuscript and to generate all figures is available at https://github.com/stoufferlab/dimensionality-of-competition and archived in Zenodo at 10.5281/zenodo.21155119^79^.

## References

[1] Gause, G. F. *The Struggle for Existence* (Williams & Wilkins Co., 1934).

[2] Birch, L. C. The meanings of competition. *Am. Nat.* **91**, 5–18 (1957).

[3] Armstrong, R. A. & McGehee, R. Competitive exclusion. *Am. Nat.* **115**, 151–170 (1980).

[4] Huisman, J. & Weissing, F. J. Biodiversity of plankton by species oscillations and chaos. *Nature* **402**, 407–410 (1999).

[5] Reich, P. B. et al. Do species and functional groups differ in acquisition and use of C, N and water under varying atmospheric CO_2_ and N availability regimes? A field test with 16 grassland species. *New Phytol.* **150**, 435–448 (2001).

[6] Craine, J. M. & Dybzinski, R. Mechanisms of plant competition for nutrients, water and light. *Funct. Ecol.* **27**, 833–840 (2013).

[7] MacArthur, R. & Levins, R. The limiting similarity, convergence, and divergence of coexisting species. *Am. Nat.* **101**, 377–385 (1967).

[8] Tilman, D. *Resource Competition and Community Structure* (Princeton University Press, 1982). 7162524

[9] Haerter, J. O., Mitarai, N. & Sneppen, K. Phage and bacteria support mutual diversity in a narrowing staircase of coexistence. *ISME J.* **8**, 2317–2326 (2014). 24858781 10.1038/ismej.2014.80 PMC4992086

[10] Chesson, P. MacArthur’s consumer-resource model. *Theor. Popul. Biol.* **37**, 26–38 (1990).

[11] Abrams, P. A. & Rueffler, C. Coexistence and limiting similarity of consumer species competing for a linear array of resources. *Ecology* **90**, 812–822 (2009). 19341150 10.1890/08-0446.1

[12] Blonder, B., Lamanna, C., Violle, C. & Enquist, B. J. The n-dimensional hypervolume. *Global Ecol. Biogeogr.* **23**, 595–609 (2014).

[13] Kraft, N. J. B., Godoy, O. & Levine, J. M. Plant functional traits and the multidimensional nature of species coexistence. *Proc. Natl. Acad. Sci. USA* **112**, 797–802 (2015). 25561561 10.1073/pnas.1413650112 PMC4311865

[14] MacArthur, R. Species packing and competitive equilibrium for many species. *Theor. Popul. Biol.* **1**, 1–11 (1970). 5527624 10.1016/0040-5809(70)90039-0

[15] Skwara, A., Lemos-Costa, P., Miller, Z. R. & Allesina, S. Modelling ecological communities when composition is manipulated experimentally. *Methods Ecol. Evol.* **14**, 696–707 (2023).

[16] Adler, P. B. et al. Competition and coexistence in plant communities: intraspecific competition is stronger than interspecific competition. *Ecol. Lett.* **21**, 1319–1329 (2018). 29938882 10.1111/ele.13098

[17] Broekman, M. J. E. et al. Signs of stabilisation and stable coexistence. *Ecol. Lett.* **22**, 1957–1975 (2019). 31328414 10.1111/ele.13349

[18] Chesson, P. L. Coexistence of competitors in a stochastic environment: the storage effect. In *Population Biology* (eds. Freedman, H. I. & Strobeck, C.) 188–198 (Springer-Verlag, 1983).

[19] Chesson, P. L. The storage effect in stochastic population models. In *Mathematical Ecology* (eds. Levin, S. A. & Hallam, T. G.) 76–89 (Springer-Verlag, 1984).

[20] Adler, P. B., HilleRisLambers, J. & Levine, J. M. A niche for neutrality. *Ecol. Lett.* **10**, 95–104 (2007). 17257097 10.1111/j.1461-0248.2006.00996.x

[21] Carmel, Y. et al. Using exclusion rate to unify niche and neutral perspectives on coexistence. *Oikos* **126**, 1451–1458 (2017).

[22] Tilman, D. Resource competition between plankton algae: an experimental and theoretical approach. *Ecology* **58**, 338–348 (1977).

[23] Chesson, P. Mechanisms of maintenance of species diversity. *Annu. Rev. Ecol. Syst.* **31**, 343–366 (2000).

[24] Allesina, S. & Levine, J. M. A competitive network theory of species diversity. *Proc. Natl. Acad. Sci. USA* **108**, 5638–5642 (2011). 21415368 10.1073/pnas.1014428108 PMC3078357

[25] Saavedra, S. et al. A structural approach for understanding multispecies coexistence. *Ecol. Monogr.* **87**, 470–486 (2017).

[26] Goldberg, D. E. Competitive ability: definitions, contingency and correlated traits. *Philos. Trans. R. Soc. Lond. B* **351**, 1377–1385 (1996).

[27] Silvertown, J. Plant coexistence and the niche. *Trends Ecol. Evol.* **19**, 605–611 (2004).

[28] Levine, J. M. & HilleRisLambers, J. The importance of niches for the maintenance of species diversity. *Nature* **461**, 254–257 (2009). 19675568 10.1038/nature08251

[29] Grover, J. P. *Resource Competition* (Chapman & Hall, 1997).

[30] Terry, J. C. D., Chen, J. & Lewis, O. T. Natural enemies have inconsistent impacts on the coexistence of competing species. *J. Anim. Ecol.* **90**, 2277–2288 (2021). 34013519 10.1111/1365-2656.13534

[31] Johnson, C. A., Dutt, P. & Levine, J. M. Competition for pollinators destabilizes plant coexistence. *Nature* **607**, 721–725 (2022). 35859181 10.1038/s41586-022-04973-x

[32] Godoy, O., Kraft, N. J. B. & Levine, J. M. Phylogenetic relatedness and the determinants of competitive outcomes. *Ecol. Lett.* **17**, 836–844 (2014). 24766326 10.1111/ele.12289

[33] Lemos-Costa, P., Miller, Z. R. & Allesina, S. Phylogeny structures species’ interactions in experimental ecological communities. *Ecol. Lett.* **27**, e14490 (2024). 39152685 10.1111/ele.14490

[34] Grime, J. P. & Pierce, S. *The Evolutionary Strategies that Shape Ecosystems* (John Wiley & Sons, Inc., 2012).

[35] Goldberg, D. E. & Landa, K. Competitive effect and response: hierarchies and correlated traits in the early stages of competition. *J. Ecol.* **79**, 1013–1030 (1991).

[36] Goldberg, D. E. Components of resource competition in plant communities. In *Perspectives in Plant Competition* (eds. Grace, J. & Tilman, D.) 27–49 (Academic Press, Inc., 1990).

[37] Hart, S. P., Freckleton, R. P. & Levine, J. M. How to quantify competitive ability. *J. Ecol.* **106**, 1902–1909 (2018).

[38] Bimler, M. D., Mayfield, M. M., Martyn, T. E. & Stouffer, D. B. Estimating interaction strengths for diverse horizontal systems using performance data. *Methods Ecol. Evol.* **14**, 968–980 (2023).

[39] Pianka, E. R. Niche overlap and diffuse competition. *Proc. Natl. Acad. Sci. USA* **71**, 2141–2145 (1974). 4525324 10.1073/pnas.71.5.2141 PMC388403

[40] Pavoine, S., Vela, E., Gachet, S., de Bélair, G. & Bonsall, M. B. Linking patterns in phylogeny, traits, abiotic variables and space: a novel approach to linking environmental filtering and plant community assembly. *J. Ecol.* **99**, 165–175 (2011).

[41] Kinlock, N. L. A meta-analysis of plant interaction networks reveals competitive hierarchies as well as facilitation and intransitivity. *Am. Nat.* **194**, 640–653 (2019). 31613666 10.1086/705293

[42] Bimler, M. D., Stouffer, D. B., Martyn, T. E. & Mayfield, M. M. Plant interaction networks reveal the limits of our understanding of diversity maintenance. *Ecol. Lett.* **27**, e14376 (2024). 38361464 10.1111/ele.14376

[43] Dalla Riva, G. V. & Stouffer, D. B. Exploring the evolutionary signature of food webs’ backbones using functional traits. *Oikos* **125**, 446–456 (2016).

[44] Shepard, R., Brozell, S. R. & Gidofalvi, G. The representation and parametrization of orthogonal matrices. *J. Phys. Chem. A* **119**, 7924–7939 (2015). 25946418 10.1021/acs.jpca.5b02015

[45] Jauch, M., Hoff, P. D. & Dunson, D. B. Random orthogonal matrices and the Cayley transform. *Bernoulli* **26**, 1560–1586 (2020).

[46] Hofbauer, J. & Sigmund, K. *Evolutionary Games and Population Dynamics* (Cambridge University Press, 1998).

[47] Cattell, R. B. The scree test for the number of factors. *Multivar. Behav. Res.* **1**, 245–276 (1966). 10.1207/s15327906mbr0102_10 26828106

[48] Zhu, M. & Ghodsi, A. Automatic dimensionality selection from the scree plot via the use of profile likelihood. *Comp. Stat. Data Anal.* **51**, 918–930 (2006).

[49] Godoy, O., Stouffer, D. B., Kraft, N. J. B. & Levine, J. M. Intransitivity is infrequent and fails to promote annual plant coexistence without pairwise niche differences. *Ecology* **98**, 1193–1200 (2017). 28241383 10.1002/ecy.1782

[50] Petry, W. K., Kandlikar, G. S., Kraft, N. J. B., Godoy, O. & Levine, J. M. A competition-defence trade-off both promotes and weakens coexistence in an annual plant community. *J. Ecol.* **106**, 1806–1818 (2018).

[51] Matías, L., Godoy, O., Gómez-Aparicio, L. & Pérez-Ramos, I. M. An experimental extreme drought reduces the likelihood of species to coexist despite increasing intransitivity in competitive networks. *J. Ecol.* **106**, 826–837 (2018).

[52] Araújo, M. B. & & Rahbek, C. How does climate change affect biodiversity?. *Science* **313**, 1396 (2006). 16959994 10.1126/science.1131758

[53] Dwyer, J. M., Hobbs, R. J., Wainwright, C. E. & Mayfield, M. M. Climate moderates release from nutrient limitation in natural annual plant communities. *Global Ecol. Biogeogr.* **24**, 549–561 (2015).

[54] Colwell, R. K. & Rangel, T. F. Hutchinson’s duality: the once and future niche. *Proc. Natl. Acad. Sci. USA* **106**, 19651 (2009). 19805163 10.1073/pnas.0901650106 PMC2780946

[55] Díaz, S. et al. The global spectrum of plant form and function. *Nature* **529**, 167–171 (2016). 26700811 10.1038/nature16489

[56] Cohen, D. Optimizing reproduction in a randomly varying environment. *J. Theor. Biol.* **12**, 119–129 (1966). 6015423 10.1016/0022-5193(66)90188-3

[57] Watkinson, A. R. Density-dependence in single-species populations of plants. *J. Theor. Biol.* **83**, 345–357 (1980).

[58] McPeek, M. A. Mechanisms influencing the coexistence of multiple consumers and multiple resources: resource and apparent competition. *Ecol. Monogr.* **89**, e01328 (2019).

[59] Daniel, C., Allan, E., Saiz, H. & Godoy, O. Fast-slow traits predict competition network structure and its response to resources and enemies. *Ecol. Lett.* **27**, e14425 (2024). 38577899 10.1111/ele.14425

[60] Laughlin, D. C. The intrinsic dimensionality of plant traits and its relevance to community assembly. *J. Ecol.* **102**, 186–193 (2014).

[61] Wainwright, C. E., HilleRisLambers, J., Lai, H. R., Loy, X. & Mayfield, M. M. Distinct responses of niche and fitness differences to water availability underlie variable coexistence outcomes in semi-arid annual plant communities. *J. Ecol.* **107**, 293–306 (2019).

[62] Bowler, C. H., Weiss-Lehman, C., Towers, I. R., Mayfield, M. & Shoemaker, L. G. Accounting for demographic uncertainty increases predictions for species coexistence: a case study with annual plants. *Ecol. Lett.* **25**, 1618–1628 (2022). 35633300 10.1111/ele.14011 PMC9328198

[63] Stouffer, D. B., Wainwright, C. E., Flanagan, T. & Mayfield, M. M. Cyclic population dynamics and density-dependent intransitivity as pathways to coexistence between co-occurring annual plants. *J. Ecol.* **106**, 838–851 (2018).

[64] Holt, R. D. Bringing the Hutchinsonian niche into the 21st century: ecological and evolutionary perspectives. *Proc. Natl. Acad. Sci. USA* **106**, 19659–19665 (2009). 19903876 10.1073/pnas.0905137106 PMC2780934

[65] Barbier, M., de Mazancourt, C., Loreau, M. & Bunin, G. Fingerprints of high-dimensional coexistence in complex ecosystems. *Phys. Rev. X* **11**, 011009 (2021).

[66] Hutchinson, G. E. Concluding remarks. Population Studies: Animal Ecology and Demography.* Cold Spring Harbor Symposia on Quantitative Biology* **22**, 415–427 (1957).

[67] Keddy, P. A. Assembly and response rules: two goals for predictive community ecology. *J. Veg. Sci.* **3**, 157–164 (1992).

[68] Laughlin, D. C., Joshi, C., van Bodegom, P. M., Bastow, Z. A. & Fulé, P. Z. A predictive model of community assembly that incorporates intraspecific trait variation. *Ecol. Lett.* **15**, 1291–1299 (2012). 22906233 10.1111/j.1461-0248.2012.01852.x

[69] Bimler, M. D., Stouffer, D. B., Lai, H. R. & Mayfield, M. M. Accurate predictions of coexistence in natural systems require the inclusion of facilitative interactions and environmental dependency. *J. Ecol.* **106**, 1839–1852 (2018).

[70] Song, C., Rohr, R. P. & Saavedra, S. A guideline to study the feasibility domain of multi-trophic and changing ecological communities. *J. Theor. Biol.* **450**, 30–36 (2018). 29702110 10.1016/j.jtbi.2018.04.030

[71] Van Dyke, M. N., Levine, J. M. & Kraft, N. J. B. Small rainfall changes drive substantial changes in plant coexistence. *Nature* **611**, 507–511 (2022). 36323782 10.1038/s41586-022-05391-9

[72] Cervantes-Loreto, A. et al. Environmental context, parameter sensitivity, and structural sensitivity impact predictions of annual-plant coexistence. *Ecol. Monogr.* **93**, e1592 (2023).

[73] Mayfield, M. M. Higher-order interactions capture unexplained complexity in diverse communities. *Nature Ecol. Evol.* **1**, 0062 (2017). 10.1038/s41559-016-0062 28812740

[74] Rao, C., Toutenberg, H., Shalabh & Heumann, C. *Linear Models and Generalizations* (Springer, 2010).

[75] Bolker, B. & R Development Core Team. *bbmle: Tools for General Maximum Likelihood Estimation* https://CRAN.R-project.org/package=bbmle (R Package Version 1.0.25.1, 2023).

[76] R Core Team. *R: A Language and Environment for Statistical Computing* http://www.R-project.org (R Foundation for Statistical Computing).

[77] Legendre, P. & Legendre, L. *Numerical Ecology* (Elsevier, 2012).

[78] Oksanen, J. et al. *vegan: Community Ecology Package* https://CRAN.R-project.org/package=vegan (R Package Version 2.7-3, 2026).

[79] Stouffer, D. B., Godoy, O., Dalla Riva, G. V. & Mayfield, M. M. Code for: the dimensionality of plant–plant competition. *Zenodo*10.5281/zenodo.21155119 (2026).10.1038/s41467-026-76980-942649222

[80] Svenning, J. C., Fabbro, T. & Wright, S. J. Seedling interactions in a tropical forest in Panama. *Oecologia* **155**, 143–150 (2008). 17965886 10.1007/s00442-007-0884-y

[81] Gao, S. et al. Plant interactions with changes in coverage of biological soil crusts and water regime in Mu Us Sandland, China. *PLoS ONE* **9**, e87713 (2014). 24498173 10.1371/journal.pone.0087713 PMC3909207

[82] Costa, C. S., Marangoni, J. C. & Azevedo, A. M. Plant zonation in irregularly flooded salt marshes: relative importance of stress tolerance and biological interactions. *J. Ecol.* **91**, 951–965 (2003).

[83] Domènech, R. & Vilà, M. Response of the invader *Cortaderia selloana* and two coexisting natives to competition and water stress. *Biol. Invasions* **10**, 903–912 (2008).

[84] Farrer, E. C. & Goldberg, D. E. Patterns and mechanisms of conspecific and heterospecific interactions in a dry perennial grassland. *J. Ecol.* **99**, 265–276 (2011).

[85] Gurevitch, J., Wilson, P., Stone, J. L., Teese, P. & Stoutenburgh, R. J. Competition among old-field perennials at different levels of soil fertility and available space. *J. Ecol.* **78**, 727–744 (1990).

[86] Hedberg, A. M., Borowicz, V. A. & Armstrong, J. E. Interactions between a hemiparasitic plant, Pedicularis canadensis L. (Orobanchaceae), and members of a tallgrass prairie community. *J. Torrey Bot. Soc.* **132**, 401–410 (2005).

[87] Weigelt, A., Steinlein, T. & Beyschlag, W. Does plant competition intensity rather depend on biomass or on species identity?. *Basic Appl. Ecol.* **3**, 85–94 (2002).

