# Supplementary material for: The dimensionality of plant–plant competition
Source: Nat Commun. 2026 Aug 26;17:9065. doi: 10.1038/s41467-026-76980-9 (PMC13518944; doi:10.1038/s41467-026-76980-9)
Supplement: Supplementary file 1 [file 41467_2026_76980_MOESM1_ESM.pdf]

SUPPLEMENTARY INFORMATION

The dimensionality of plant–plant competition

Stouffer, Godoy, Dalla Riva, & Mayfield

Supplementary Note 1

Interaction coefficients resulting from consumer–resource models

We study here response–effect parameterized interactions in the context of "phenomenological" models where the impacts of interactions are modulated by species' densities[1–3]. To better contextualize what could give rise to species showing different "response traits" and different "effect traits", it is also helpful to see how interactions emerge from a more "mechanistic" model within which species compete over explicit resources. One such model is that proposed by MacArthur[4] with consumer dynamics given by

$$\frac{1}{N_i}\frac{dN_i}{dt} = \sum_{k=1}^{d} w_{ik} c_{ik} X_k - m_i \tag{S1}$$

where N_i is the density of consumer species i, X_k is the density of resource k, c_{ik} is the resource utilization rate of resource k by consumer i, w_{ik} is the "value" of resource k to consumer i, and m_i is the mortality rate of consumer i; and resource dynamics show logistic population growth according to

$$\frac{1}{X_k}\frac{dX_k}{dt} = \rho_k \left(1 - \frac{X_k}{K_k}\right) - \sum_{j=1}^{S} c_{jk} N_j \,, \tag{S2}$$

where ρ_k is the growth rate of resource k, and K_k is the carrying capacity of resource k. If the timescale of resource dynamics is very fast relative to that of consumers[5], then we can approximate the resource density at every moment in time as its consumer-dependent equilibrium value (X_k^*). For each resource, this is given by

$$X_k^* = K_k - \frac{K_k}{\rho_k} \sum_{j=1}^{S} c_{jk} N_j \, . \tag{S3}$$

Since all X_k^* must be greater than or equal to zero, the parameter combination K_k/ρ_k gives us an indication of how limiting each of the different resources is. That is, when $K_k/\rho_k \approx 0$, resource k is expected to always be near its carrying capacity K_k and thus maximally available to all consumers; in contrast, as K_k/ρ_k increases away from 0, resource k becomes more and more limiting because its equilibrium density will get closer and closer to 0.

If we substitute these equilibrium resource densities into Eq. (S1), the expression for consumer dynamics can be rewritten as

$$\frac{1}{N_i}\frac{dN_i}{dt} = \beta_i - \sum_{j=1}^{S} a_{ij|d}\, N_j \, , \tag{S4}$$

where the interaction coefficients $a_{ij|d}$ are each given by

$$a_{ij|d} = \sum_{k=1}^{d} \frac{K_k}{\rho_k} w_{ik} c_{ik} c_{jk} \, . \tag{S5}$$

When species compete over explicit resources, species–species interactions are therefore driven by the resource dynamics (as influenced by K_k/ρ_k), a focal species' ability to exploit *and make use of* those resources (as influenced by $w_{ik}c_{ik}$), and a competitor's ability to exploit those resources (as influenced by c_{jk}).

Similar to our response–effect framework, Eq. (S5) implies that the interaction strengths that emerge from such a model of consumer–resource dynamics can also be decomposed

into a sum of different contributions across the d dimensions corresponding to the resources over which species compete (as in Eq. (1) from the main text; see also Section Supplementary Note 3 below). Note, however, that even if $d > S$ the rank of interaction matrix consisting of the coefficients $a_{ij|d}$ will never be greater than S. This implies that any consumer–resource system described with Eq. (S4) and with $d > S$ resources can always be re-represented mathematically as a system with only $d = S$ resources. In contrast, the interaction matrix consisting of the coefficients $a_{ij|d}$ will always be rank deficient whenever $d < S$. Consequently, in such a scenario a maximum of d species would be able to coexist if population dynamics are given by Eq. (S4).

Mathematically, we could rewrite Eq. (S5) in matrix form as $\mathbf{A} = \mathbf{UDV}^T$, where $\mathbf{U}$ is an $S \times d$ matrix with elements $u_{ik} = w_{ik}c_{ik}$, $\mathbf{V}$ is an $S \times d$ matrix with elements $v_{ik} = c_{ik}$, and $\mathbf{D}$ is a $d \times d$ diagonal matrix with the values $\theta_k = K_k/\rho_k$ along the diagonal. Though this also bears strong mathematical similarity to our SVD-based parameterization ($\mathbf{A} = \mathbf{R}\,\mathbf{\Sigma}\,\mathbf{E}^T$), we caution that $\mathbf{U} \neq \mathbf{R}$, $\mathbf{V} \neq \mathbf{E}$, and $\mathbf{D} \neq \mathbf{\Sigma}$. This is most directly due to the fact that our inferential approach based on singular value decomposition will seek to "realign" the emergent outcomes of Eq. (S5) such that the leading dimensions of $\mathbf{R}$ and $\mathbf{E}$ capture as much of the structure in the matrix $\mathbf{A}$ as possible, thereby turning resources X_k into latent, "effective resources" that partition distinct ecological strategies among species (as described in the main text). Indeed, this is precisely what would occur in the aforementioned situation in which $d > S$.

Supplementary Note 2

Inferred niche dimensionality of interaction coefficients resulting from consumer–resource models

As noted in the previous section, even when a matrix $\mathbf{A}$ is populated with values

from Eq. (S5) that arise from a model with d explicit resources, there may be scenarios where $\mathbf{A}$ can be well approximated by niche dimensionality $\hat{d} < d$. Consider, for example, a system with two equally limiting resources (i.e., $\theta_1 \approx \theta_2$) and where each species' utilizations of those two resources are strongly correlated (i.e., there is a strong correlation between coefficients c_{i1} and c_{i2} across all species i). In such a situation, a 1-dimensional approximation is likely to be quite accurate[5], and this accuracy will ultimately be driven by the extent to which the values of those resources across species (i.e., the coefficients w_{ik}) are or are not also similar. Indeed, this is one of the strengths of singular value decomposition that has made it useful across a variety of fields[6]: even when input data may be of high dimension, it may be possible to "compress" those data to show that their output exhibits fewer degrees of freedom than appear on the surface.

To examine this more quantitatively, we studied controlled parameterizations of Eq. (S5) for which we know the underlying number of resources d. As described in the previous section, there are essentially three potential sources of variation that could lead to $\hat{d} < d$: (1) the vector $\boldsymbol{\theta}$ of resource dynamics/resource limitation parameters (made up of d elements $\theta_k = K_k/\rho_k$); (2) the matrix $\mathbf{W}$ of resource value parameters (made up of $S \times d$ elements w_{ik}); and (3) the matrix $\mathbf{C}$ of resource utilization parameters (made up of $S \times d$ elements c_{ik}). To generate an emergent interaction matrix $\mathbf{A}$, we therefore need the set of $\{\boldsymbol{\theta}, \mathbf{W}, \mathbf{C}\}$.

Two species and two resources

For simplicity, we first focus on a two-species, two-resource scenario where $S = 2$ and $d = 2$. When the values in $\boldsymbol{\theta}$, $\mathbf{W}$, and/or $\mathbf{C}$ were allowed to vary at random, we drew them all from a uniform distribution across the interval $[0, 1]$; when those same parameters were set to be identical across resources and/or species, we fixed them to 0.5 (the mean of

the aforementioned uniform distribution). Though it was not a strict requirement specified by MacArthur[4], we constrained species' resource utilization vectors (i.e., rows in $\mathbf{C}$) to sum to 1 to remove potential impacts of their magnitudes on the niche dimensionality of the resulting interaction matrices.

With each randomly generated set of parameters ($\{\boldsymbol{\theta}, \mathbf{W}, \mathbf{C}\}$), we quantified the variation in $\boldsymbol{\theta}$ with $\Delta_\theta = |\theta_1 - \theta_2|$, which gives values between $\Delta_\theta = 0$ (both resources are equally limiting) and $\Delta_\theta = 1$ (only one resource is limiting). We quantified the variation in $\mathbf{W}$ with Δ_W given by the Bray–Curtis distance[7] across rows in $\mathbf{W}$, which gives values between $\Delta_W = 0$ (no variation across species) and $\Delta_W = 1$ (the species obtain value from completely different resources). We quantified the variation in $\mathbf{C}$ with Δ_C given by the Bray–Curtis distance across rows in $\mathbf{C}$, which gives values between $\Delta_C = 0$ (no variation across species) and $\Delta_C = 1$ (the species utilize completely different resources). We can thus use the set of $\{\Delta_\theta, \Delta_W, \Delta_C\}$ as estimates of the amount of "input variation" for a given parameterization.

Lastly, we used Eq. (S5) to calculate the interaction matrix $\mathbf{A}$ for each randomly generated set of parameters ($\{\boldsymbol{\theta}, \mathbf{W}, \mathbf{C}\}$), and then performed an SVD with that matrix. As described in the Methods, we estimated the variance explained by the first niche dimension as $\nu = \sigma_1^2 / \sum_{k=1}^{2} \sigma_k^2$, where the σ_k are the singular values of the interaction matrix $\mathbf{A}$. Values of $\nu = 1$ indicate that the first niche dimension explains all interaction variation (i.e., $\hat{d} = 1$), and values of $\nu = 0.5$ indicate that both niche dimensions have equivalent explanatory power (i.e., $\hat{d} = 2$). The value ν is thus a quantitative indicator of the "output" niche dimensionality of the emergent interaction matrix. We compared these estimates of ν to different amounts of input variation in $\{\Delta_\theta, \Delta_W, \Delta_C\}$ to better disentangle drivers of low and high output: niche dimensionality and the effective number of resources underpinning the matrix $\mathbf{A}$.

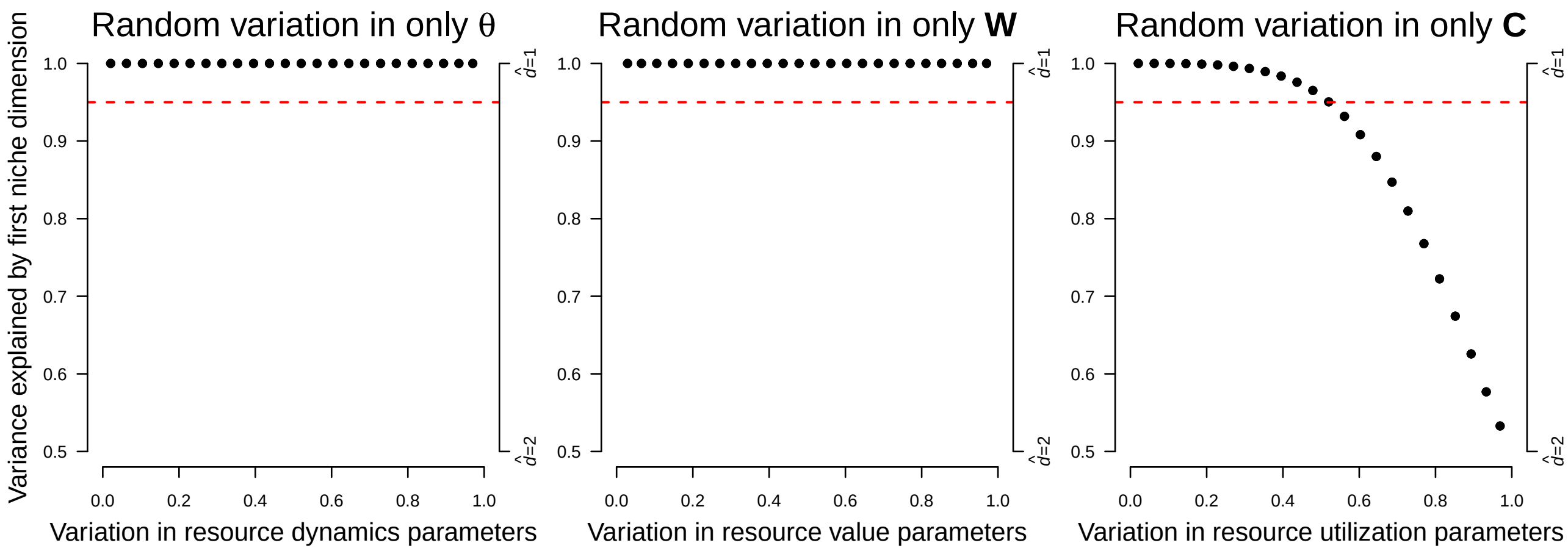

Figure S1: Niche dimensionality of interaction matrices generated by MacArthur's consumer–resource model with two species ($S = 2$) and two resources ($d = 2$). (Left panel) We show the mean amount of variance explained by the first singular value for 10^6 interaction matrices $\mathbf{A}$ generated using Eq. (S5) and for which only resource dynamics parameters ($\boldsymbol{\theta}$) were allowed to vary at random. The alternative y-axis indicates the niche dimensionality, and our approach would conclude that any matrix whose value is above the dashed red line is adequately captured by niche dimensionality $\hat{d} = 1$. (Middle panel) We show the same but for 10^6 interaction matrices $\mathbf{A}$ generated using Eq. (S5) and for which only resource value parameters ($\mathbf{W}$) were allowed to vary at random. (Right panel) We show the same but for 10^6 interaction matrices $\mathbf{A}$ generated using Eq. (S5) and for which only resource utilization parameters ($\mathbf{C}$) were allowed to vary at random.

We first explored situations within which only one source of parameter variation was included at a time (e.g., $\{\Delta_\theta \geq 0, \Delta_W = 0, \Delta_C = 0\}$). Here, we observed that the resource utilization matrix $\mathbf{C}$ was the only type of parameter that, in isolation, was able to influence the inferred niche dimensionality in the resulting interaction matrix $\mathbf{A}$ (Fig. S1). This makes mathematical sense as the matrix $\mathbf{A}$ arising from Eq. (S5) will always be rank 1 unless there is some variation across species in $\mathbf{C}$. As a result, when the resource utilization parameters of the two resources run parallel to each other ($\Delta_C = 0$), the singular value of the first niche dimension is easily able to capture more than 95% of the variation in the matrix $\mathbf{A}$. Indeed, at the extreme of $\Delta_C = 0$, the two species are

ecologically equivalent[5,8] and thus niche dimensionality is low because mathematically there is actually only one "type" of species. As the difference between species in resource utilization parameters increases (i.e., as Δ_C approaches 1), so too does the probability that our approach would argue that the effective niche dimensionality is $\hat{d} = 2$ for such randomly parameterized assemblages.

Variation in $\mathbf{C}$ thus plays a critical role in the inferred niche dimensionality of the interaction matrix $\mathbf{A}$. As described in the main text (and elsewhere[5]), this implies that the more the resource utilization of any two or more species in an observed assemblage resemble each other, the less likely we are to require both niche dimensions to capture the resulting patterns of interactions. However, real-world systems are unlikely to have variation in resource utilization ($\Delta_C > 0$) but (i) no variation in resource dynamics ($\Delta_\theta = 0$) or (ii) no variation in how different species value different resources ($\Delta_W = 0$). We thus next investigated the extent to which random variation in $\boldsymbol{\theta}$ and $\mathbf{W}$ were capable of creating parameterized matrices $\mathbf{A}$ with an effective niche dimensionality of $\hat{d} = 1$ even when the resource utilization was very different across species. To do so, we set $\mathbf{C}$ to be equal to a matrix with values of 0.95 along the diagonal and values of 0.05 off diagonal, which corresponds to $\Delta_C = 0.9$. We considered three scenarios for how $\boldsymbol{\theta}$ and $\mathbf{W}$ could be parameterized. First, we allowed for random variation in $\boldsymbol{\theta}$ but set $\mathbf{W}$ to be identical across species (i.e., all $w_{ik} = 0.5$); second, we allowed for random variation in $\mathbf{W}$ but set $\boldsymbol{\theta}$ to be identical across resources (i.e., all $\theta_k = 0.5$); and third, we allowed for random variation in both $\boldsymbol{\theta}$ and $\mathbf{W}$.

In the first scenario, we found that as Δ_θ increased, so too did the variance in $\mathbf{A}$ captured by the singular value of the first niche dimension (Fig. S2). At very low variation in $\boldsymbol{\theta}$, essentially 100% of the randomly generated matrices had full dimensionality ($\hat{d} = 2$). At this end of the spectrum, both niche dimensions are necessary in order to fully capture

the differences in interactions across species (Fig. S2**a**), and this is due to the fact that both resources can be thought of as "equally limiting". At sufficiently high variation in $\boldsymbol{\theta}$, in contrast, the first singular value essentially always explained $> 95\%$ of the observed variance in $\mathbf{A}$ indicating that the interaction matrices were low dimensional ($\hat{d} = 1$). As Δ_θ approaches 1, the interactions between species are driven by the fact that only one of the resources is truly "limiting", and thus the effective niche dimensionality of the matrix $\mathbf{A}$ is lower than the number of resources in the system (Fig. S2**d**). Therefore, while both species adopt different ecological strategies (as determined by the variation in the matrix $\mathbf{C}$), the relative ubiquity of one resource combined with the relative scarcity

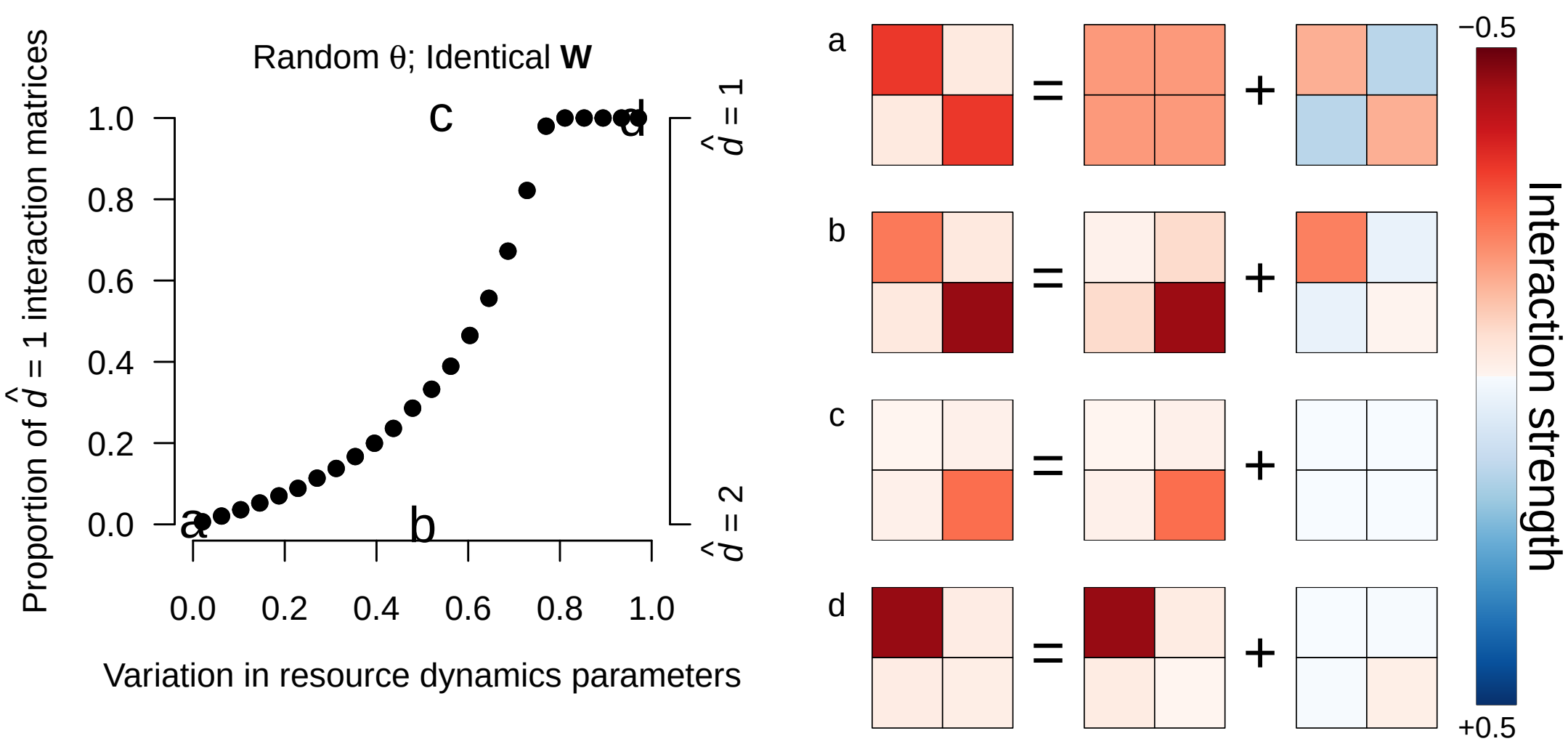

Figure S2: Niche dimensionality of interaction matrices $\mathbf{A}$ generated by MacArthur's consumer–resource model with two species ($S = 2$) and two resources ($d = 2$). We generated 10^6 matrices for which species were very different in terms of resource utilization ($\mathbf{C} = \begin{bmatrix} 0.95 & 0.05 \\ 0.05 & 0.95 \end{bmatrix}$), obtained identical values from both resources (i.e., all $w_{ik} = 0.5$), and resource dynamics parameters ($\boldsymbol{\theta}$) were allowed to vary at random. (Left panel) We plot the probability that the variance explained by the first singular value was $>$ 95% and hence that our approach would conclude that the interaction matrices are low dimensional ($\hat{d} = 1$). (Right panels) SVD-based decomposition of four representative randomly generated interaction matrices (**a**–**d**). Matrices **a** & **b** are fully dimensional ($\hat{d} = 2$) whereas matrices **c** & **d** are low dimensional ($\hat{d} = 1$).

of the other resource makes it essentially impossible for both species to simultaneously obtain a competitive advantage.

In the second scenario, we observed that as Δ_W increased, so too did the average variance in $\mathbf{A}$ captured by the singular value of the first niche dimension (Fig. S3). However, the impact of Δ_W was more muted than the impact of Δ_θ uncovered in the first scenario. When the values in $\mathbf{W}$ are allowed to vary at random, the matrices $\mathbf{A}$ generated at random could appear both high dimensional ($\hat{d} = 2$) and low dimensional ($\hat{d} = 1$). At low variation in $\mathbf{W}$, the vast majority of randomly generated matrices had full dimensionality ($\hat{d} = 2$). As the amount of variation in $\mathbf{W}$ increased, the first singular value

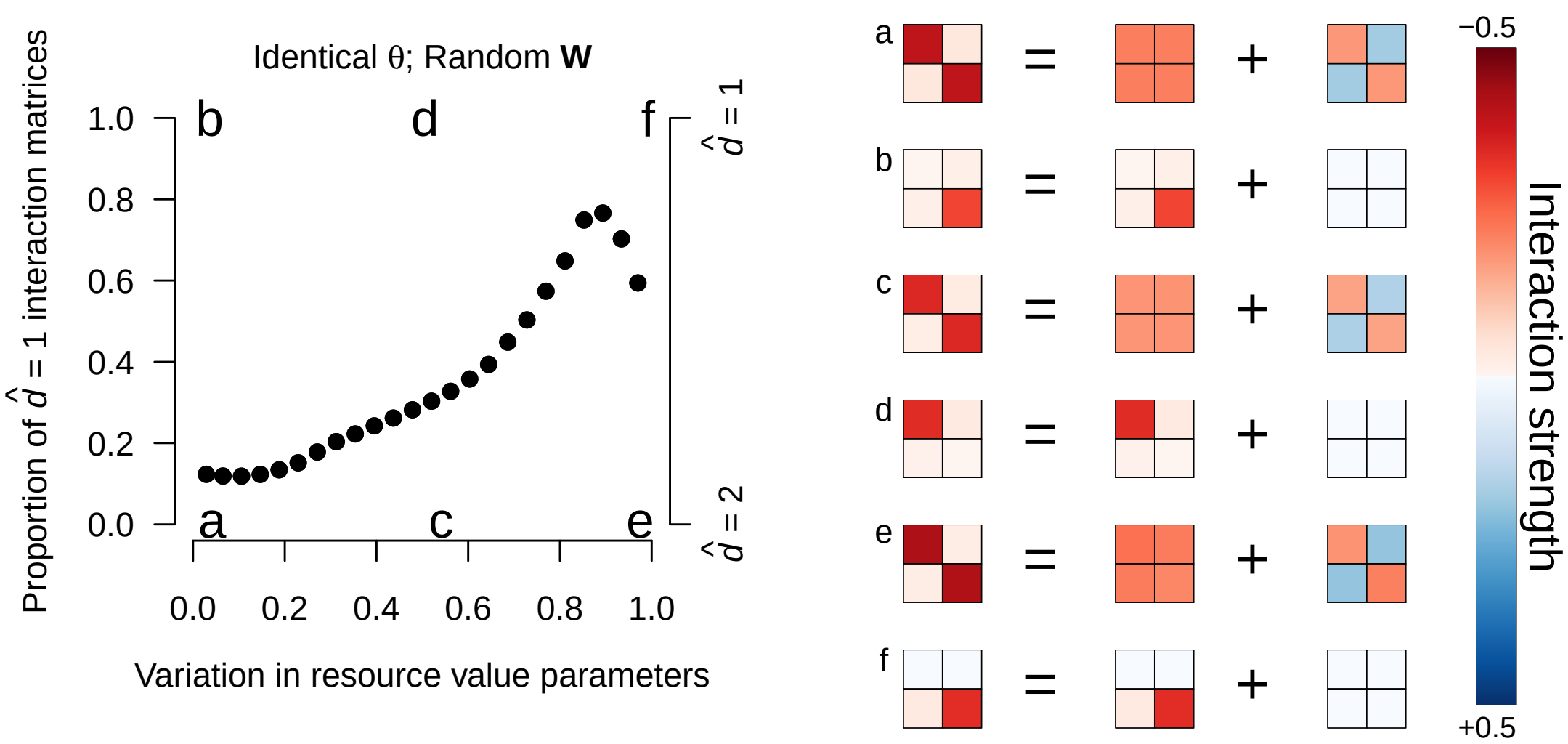

Figure S3: Niche dimensionality of interaction matrices $\mathbf{A}$ generated by MacArthur's consumer–resource model with two species ($S = 2$) and two resources ($d = 2$). We generated 10^6 matrices for which species were very distinct in terms of resource utilization ($\mathbf{C} = \begin{bmatrix} 0.95 & 0.05 \\ 0.05 & 0.95 \end{bmatrix}$), resources have equal dynamics in the environment (i.e., all $\theta_k = 0.5$), and resource value parameters ($\mathbf{W}$) were allowed to vary at random. (Left panel) We plot the probability that the variance explained by the first singular value was $> 95\%$ and hence that our approach would conclude that the interaction matrices are low dimensional ($\hat{d} = 1$). (Right panels) SVD-based decomposition of six representative randomly generated interaction matrices (**a**–**f**). Matrices **a**, **c**, & **e** are fully dimensional ($\hat{d} = 2$) whereas matrices **b**, **d**, & **f** are low dimensional ($\hat{d} = 1$).

explained $> 95\%$ of the observed variance in $\mathbf{A}$ more frequently ($\hat{d} = 1$). The interaction matrices were high dimensional when variation in $\mathbf{W}$ mirrored variation in $\mathbf{C}$ (i.e., each species obtained the greatest value from the same resource they were specialized on); they were low dimensional when variation in $\mathbf{W}$ ran opposite to or completely cancelled out variation in $\mathbf{C}$ (i.e., each species obtained the least value from the resources on which they are specialized, which may even drive many interaction coefficients toward 0). The impact of Δ_W is thus more to change the probability of randomly obtaining either of these two outcomes (Fig. S3**a**–**f**). The shift from matrices like that in Fig. S3**a** to those like in Fig. S3**f** implies that larger differences in resource value parameters can make the matrix $\mathbf{A}$ have reduced variation in interaction coefficients. Ecologically, such competitors will appear nearly identical in how they affect each other, and are unlikely to coexist[5]. One interpretation for this would be that extinction would rely on near-identical resource dynamics[9]; however, this second scenario indicates that extinction can instead by driven entirely by variation in resource value parameters overcoming the inherent benefits species would otherwise have obtained from adopting distinct resource utilization strategies.

In the third scenario, we again found that increasing both Δ_θ and Δ_W was consistent with increasing variation captured by the first singular value of the resulting matrix $\mathbf{A}$ (Fig. S4). The dependence on variation in $\boldsymbol{\theta}$ was weaker than in the first scenario and the dependence on variation in $\mathbf{W}$ was stronger than in the second scenario. We expect that this largely owes to the fact that variation in $\boldsymbol{\theta}$ could act to "cancel out" variation in $\mathbf{W}$ (and vice versa). Nevertheless, consistent with the first and second scenarios, the presence of both types of variation generally makes it harder for our approach to detect that species were different in terms of $\mathbf{C}$ or, conversely, easier for the emergent interaction matrix $\mathbf{A}$ to be low dimensional ($\hat{d} = 1$).

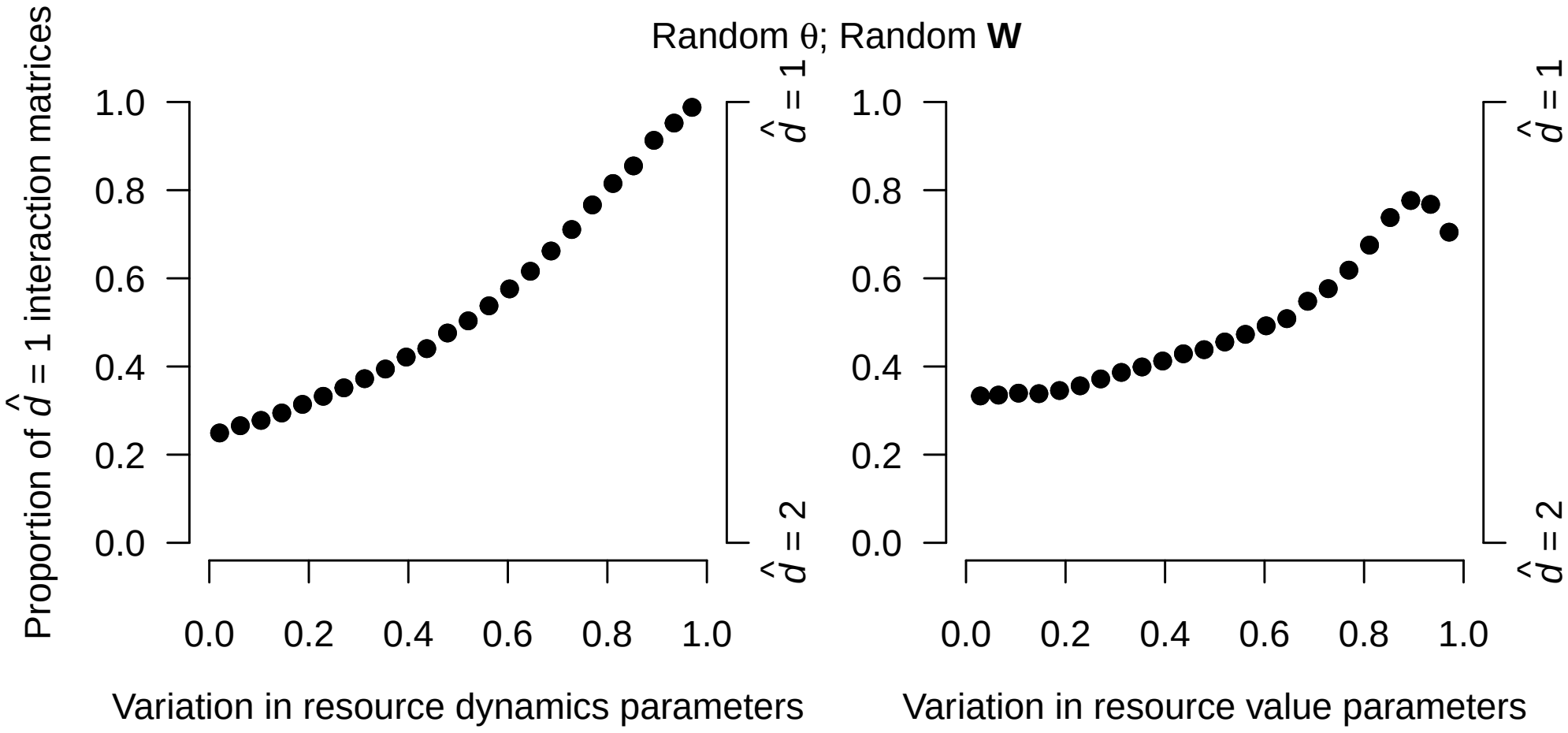

Figure S4: Niche dimensionality of interaction matrices $\mathbf{A}$ generated by MacArthur's consumer–resource model with two species ($S = 2$) and two resources ($d = 2$). We generated 10^6 matrices for which species were very distinct in terms of resource utilization ($\mathbf{C} = \begin{bmatrix} 0.95 & 0.05 \\ 0.05 & 0.95 \end{bmatrix}$), resource dynamics parameters ($\boldsymbol{\theta}$) were allowed to vary at random, and resource value parameters ($\mathbf{W}$) were allowed to vary at random. We plot the probability that the variance explained by the first singular value was $> 95\%$ as a function of Δ_θ (left panel) and as a function of Δ_W (right panel). This probability is equivalent to the extent to which our approach would conclude that the interaction matrices are low dimensional ($\hat{d} = 1$). Note that values in the left panel are averaged across variation in $\mathbf{W}$ whereas values in the right panel are averaged across variation in $\boldsymbol{\theta}$.

Ten species and ten resources

We next studied a ten-species, ten-resource scenario (i.e., where $S = 10$ and $d = 10$). Rather than study a large ensemble of randomized parameterizations, we built off the previous results to focus exclusively on how variation in $\boldsymbol{\theta}$ or in $\mathbf{C}$ influenced the emergent dimensionality of the resulting interaction matrices. Here, we explored four distinct scenarios, all of which began from the same starting point: the vector $\boldsymbol{\theta}$ was a vector of ten values $\theta_k = 0.5$ and hence all resources were equally limiting; the matrix $\mathbf{W}$ was a 10×10 matrix with all elements $w_{ik} = 0.5$, such that all resources were equally valu-

able to all species; and the matrix $\mathbf{C}$ was a 10×10 identity matrix (i.e., a matrix with 1 on the diagonal and 0's everywhere else), such that all species have unique resource utilization and thus no niche overlap. Because this starting point features (i) all resources being equally limiting and (ii) all species having unique resource utilization, the emergent interaction matrix $\mathbf{A}$ is necessarily of maximum dimensionality (i.e., $\hat{d} = 10$).

In the first scenario, we reduce the amount to which each resource is limiting on a one-by-one basis (Fig. S5). More specifically, we start from the situation where each value $\theta_k = 0.5$ for all k. We then decreased the value of the tenth resource linearly until it reached $\theta_{10} = 0.01$ (recall that the *smaller* a value of θ_k the *less* that resource is limiting), and at each value of θ_{10} we estimated the effective niche dimensionality $\hat{d}$ of the corresponding interaction matrix $\mathbf{A}$. We then repeated this process for the ninth resource, the eighth resource, and so on up until the second resource. Note that we did not decrease the value θ_1 of the first resource because doing so would return the system back to the initial conditions under which all resources were equally limiting. As expected, we found that the inferred dimensionality $\hat{d}$ very closely mirrors the number of limiting resources that are available to the different species (i.e., the number of values $\theta_k \gg 0$).

In the second, third, and fourth scenarios, we reduced the extent to which the ten hypothetical species exhibit variation in their resource utilization. Note that, with ten different species, there are myriad ways in which we could potentially "combine" a set of unique species together to reduce the underlying niche differences in the matrix $\mathbf{C}$. We therefore restricted ourselves to three scenarios which we concluded would span from more ecologically plausible to more ecologically extreme.

In the first more ecologically plausible scenario, we reduced niche differences between species in a hierarchical fashion (Figs. S6). At the early stages of the hierarchy, two species were "merged" together—from the starting point where they relied on two completely

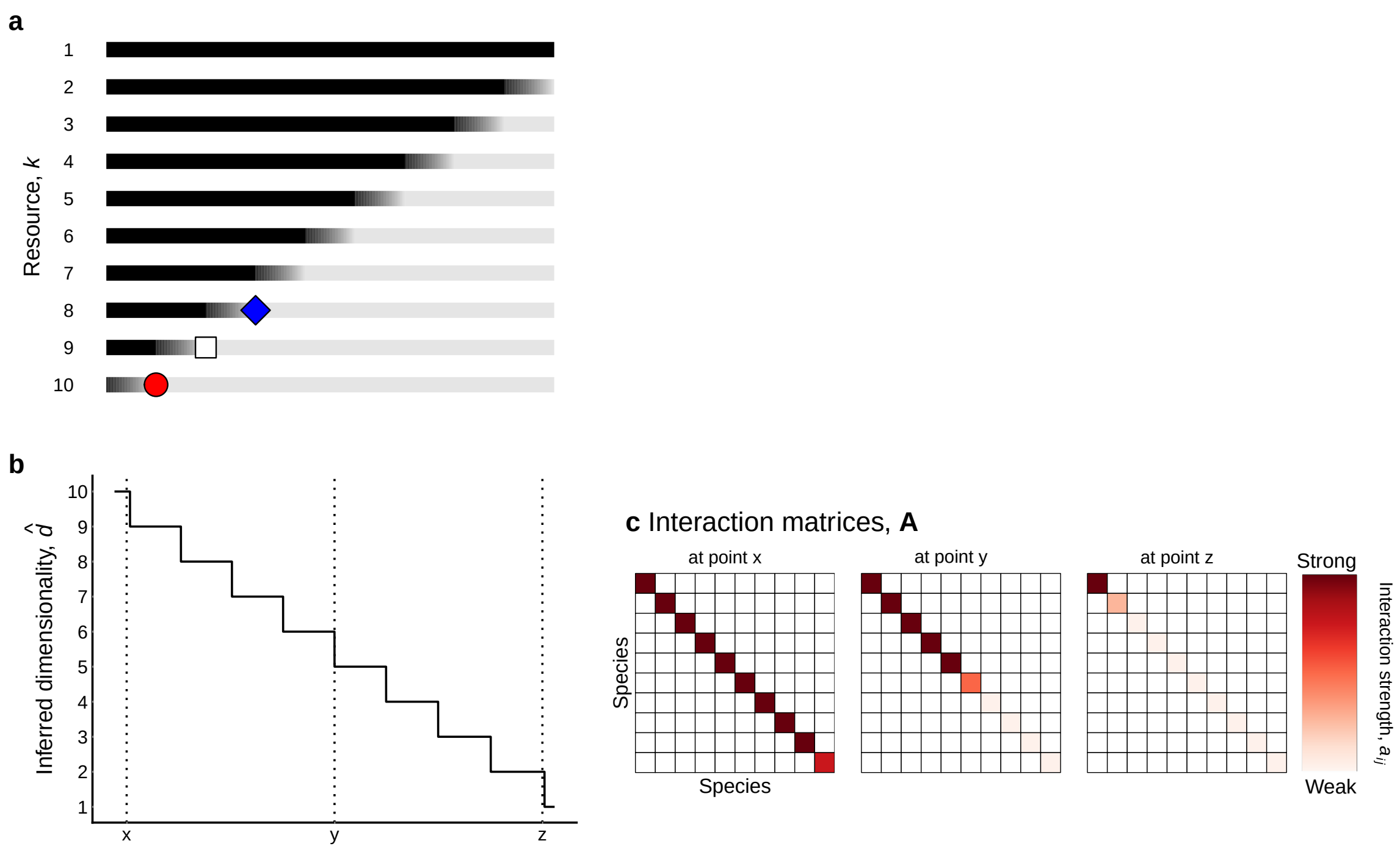

Figure S5: Niche dimensionality of interaction matrices **A** generated by MacArthur's consumer–resource model with ten species ($S = 10$) and ten resources ($d = 10$). We started from a community where species were completely different in terms of resource utilization (**C** is an identity matrix), species obtained identical values from all resources (i.e., all $w_{ik} = 0.5$), and all resource dynamics parameters were identical (i.e., all $\theta_k = 0.5$). **a**, We then systematically eliminated resources one-by-one by linearly decreasing their values to $\theta_k = 0.01$ as depicted visually here from left to right. We first eliminated the tenth resource, which reached its minimum value at the point indicated by the red circle. We next eliminated the ninth resource (whose minimum was attained as indicated by the white square), then the eighth resource (blue diamond), and so on. **b**, At each step of this process, we used our SVD-based framework to estimate the inferred dimensionality $\hat{d}$ of the resulting interaction matrix. As expected, dimensionality at the start was equal to the number of limiting resources ($\hat{d} = 10$) and decreased as fewer and fewer resources were limiting. **c**, We show the emergent interaction matrices **A** at the three points x, y, and z which are indicated with the vertical dotted lines in panel **b**. In these matrices, darker red colors indicate strong interactions and lighter colors indicate weaker interactions.

different resources to an ending point where both species used the exact same resource; further up the hierarchy, we merged these "clusters" of species together with other clusters of species, up to the point at which all species ultimately depended on the same single resource. As expected, we found that inferred niche dimensionality started at its maximal value (i.e., $\hat{d} = 10$) and decreased as species became more and more similar to each other.

In the second more ecologically plausible scenario, we also reduced niche differences between species in a hierarchical fashion (Figs. S7). However, instead of species becoming identical and specializing on the same single resource, here they became identical by generalizing on the set of resources available across all of them. As expected, we again found that inferred niche dimensionality started at its maximal value (i.e., $\hat{d} = 10$) and decreased as species became more and more similar to each other. The rate of this decline in inferred niche dimensionality was nearly identical to that seen in the previous scenario. In contrast, the primary difference between similarity across species reflecting shared specialization as opposed to shared generalization was that in the latter case the emergent interactions become comparatively weaker.

In the third, more ecologically extreme, scenario, we reduced niche differences between species in a sequential fashion such that they all converged on the *same* species in a one-by-one basis (Fig. S8). That is, we first made the second species more and more identical to the first species, until they showed 100% niche overlap; we next made the third species more and more identical to the first and second species, until they all showed 100% niche overlap with each other, and so on. In contrast to the second ten-species scenario, we found that, in the unlikely scenario that *all* species converged together to resemble a single "archetypal" species—as opposed to maintained greater within-assemblage heterogeneity—the consequential decrease in niche dimensionality can happen much more rapidly.

Similar to the two species, two resource scenario, we again observe that low dimensionality can be a by product of species exhibiting high fundamental niche variation but with unequal resource availability in the environment or many resources being available but species not fully exploiting each of these differently. The patterns observed in real-world systems are undoubtedly a result of both of these processes operating simultaneously. After all, one would need a combination of the caricature interaction matrices found across Figs. S5–S8 (as well as at least a modicum of random noise) to create something that more closely resembles the interaction matrices observed in the empirical data we study (see Section Supplementary Note 5). Moreover, other processes are also necessarily at play since the model by MacArthur [4] that we study here does not allow for the emergence of positive, facilitative interactions between species while these were occasionally observed across our empirical datasets.

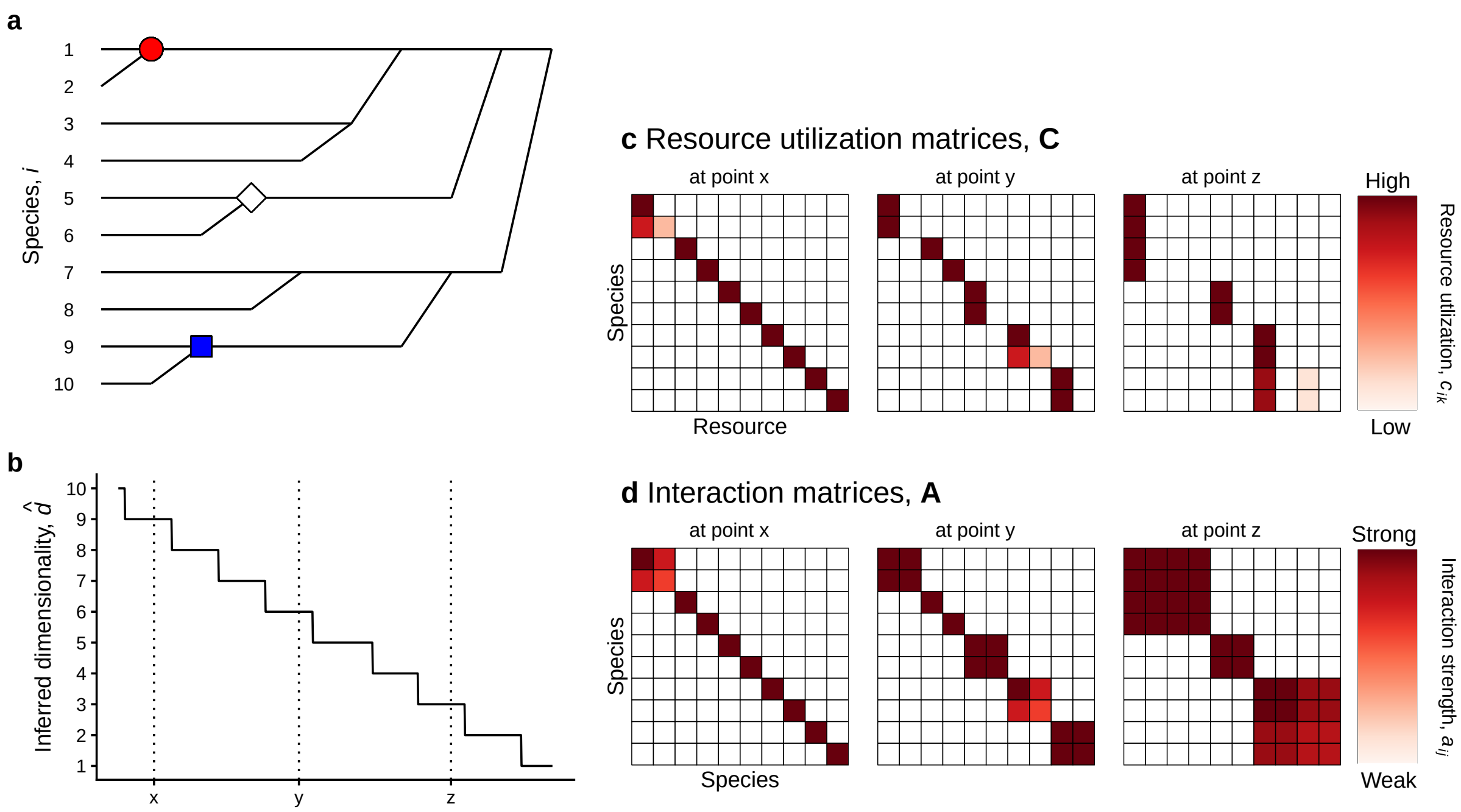

Figure S6: Niche dimensionality of interaction matrices **A** generated by MacArthur's consumer–resource model with ten species ($S = 10$) and ten resources ($d = 10$). We started from a community where species were completely different in terms of resource utilization (**C** is an identity matrix), species obtained identical values from all resources (i.e., all $w_{ik} = 0.5$), and all resource dynamics parameters were identical (i.e., all $\theta_k = 0.5$). **a**, We then systematically increased similarity across species in a stepwise and hierarchical manner as depicted from left to right in the similarity tree. We first made the second species identical to the first species, and they showed complete niche overlap at the point indicated by the red circle. Next, the tenth species was progressively made identical to the ninth species, and they became exactly identical at the point indicated by the blue square. The white diamond indicates the next merging together of species, and so on. **b**, At each step of this process, we used our SVD-based framework to estimate the inferred dimensionality $\hat{d}$ of the resulting interaction matrix. As expected, dimensionality at the start was equal to the initial number of limiting resources ($\hat{d} = 10$) and decreased as species showed increasing niche overlap. **c** & **d**, We show the underlying resource utilization matrices **C** and emergent interaction matrices **A** at the three points x, y, and z indicated with the vertical dotted lines in panel **b**. In these sets of matrices, darker red colors indicate high resource utilization or strong interactions and lighter colors indicate low resource utilization or weaker interactions, respectively.

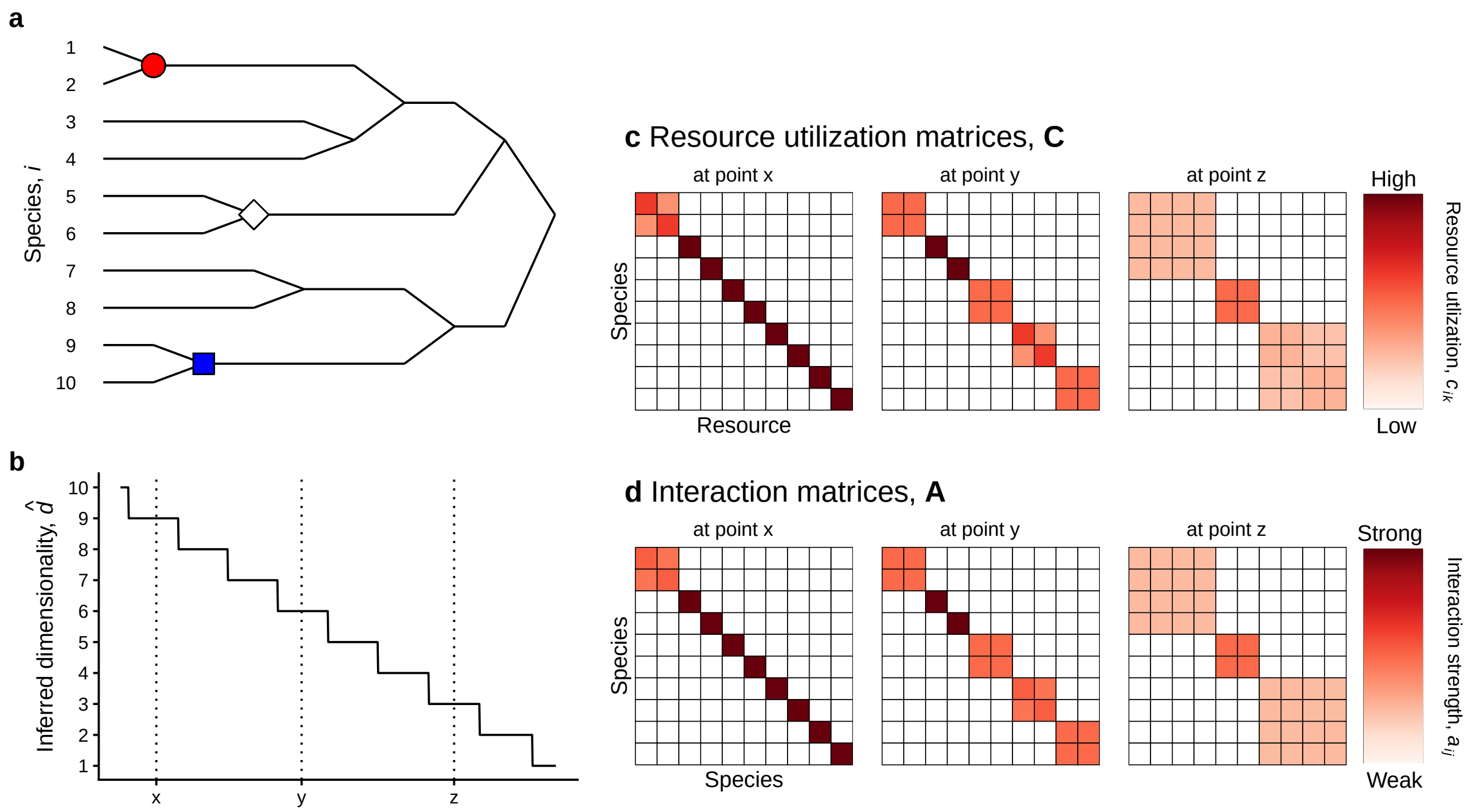

Figure S7: Niche dimensionality of interaction matrices **A** generated by MacArthur's consumer–resource model with ten species ($S = 10$) and ten resources ($d = 10$). We started from a community where species were completely different in terms of resource utilization (**C** is an identity matrix), species obtained identical values from all resources (i.e., all $w_{ik} = 0.5$), and all resource dynamics parameters were identical (i.e., all $\theta_k = 0.5$). **a**, We then systematically increased similarity across species in a stepwise and hierarchical manner as depicted from left to right in the similarity tree. We first made the first and second species identical to each other, and they showed complete niche overlap at the point indicated by the red circle. Next, the ninths and tenth species were made progressively identical to each other, and they became exactly identical at the point indicated by the blue square. The white diamond indicates the next averaging together of species, and so on. **b**, At each step of this process, we used our SVD-based framework to estimate the inferred dimensionality $\hat{d}$ of the resulting interaction matrix. As expected, dimensionality at the start was equal to the initial number of limiting resources ($\hat{d} = 10$) and decreased as species showed increasing niche overlap. **c** & **d**, We show the underlying resource utilization matrices **C** and emergent interaction matrices **A** at the three points x, y, and z indicated with the vertical dotted lines in panel **b**. In these sets of matrices, darker red colors indicate high resource utilization or strong interactions and lighter colors indicate low resource utilization or weaker interactions, respectively.

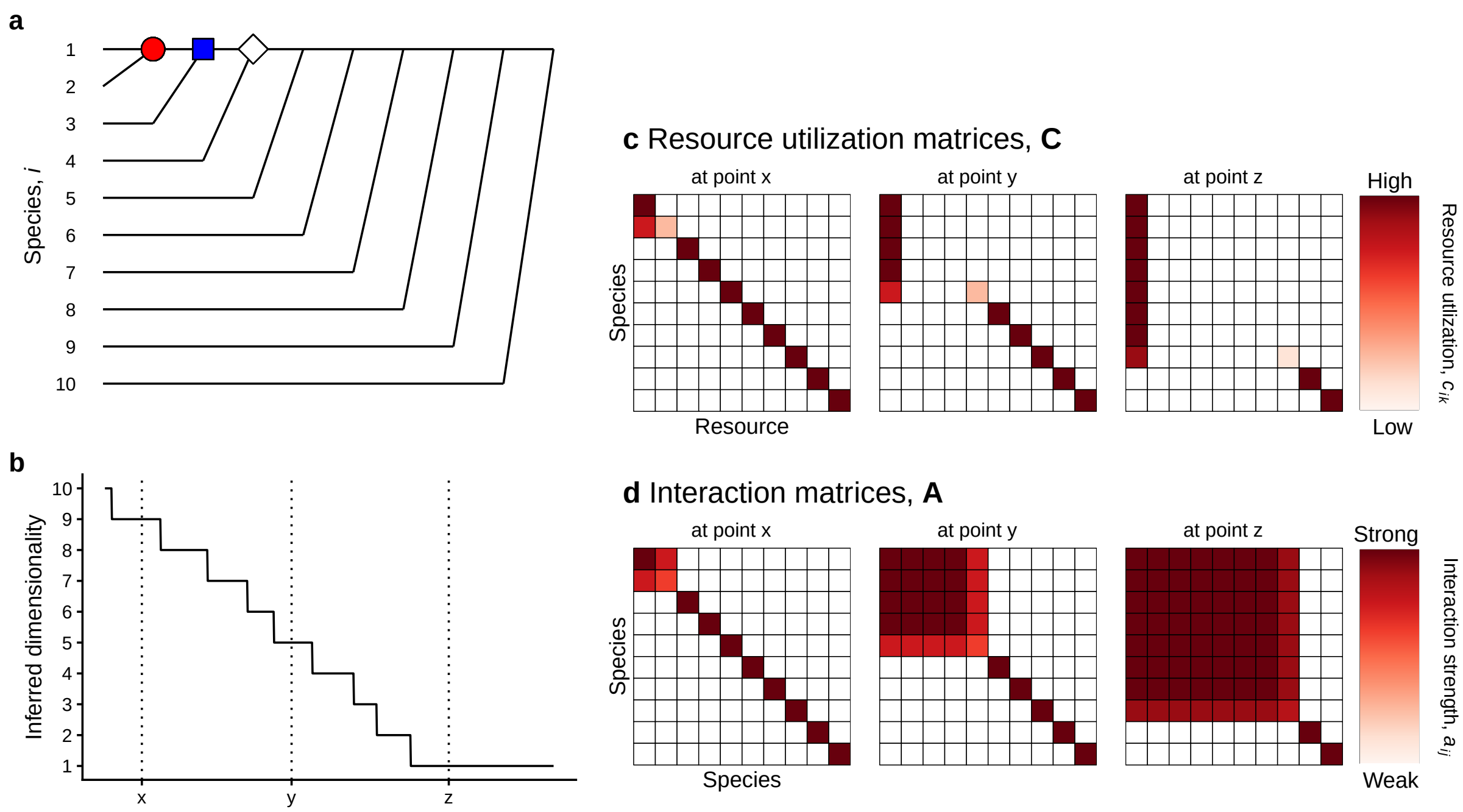

Figure S8: Niche dimensionality of interaction matrices **A** generated by MacArthur's consumer–resource model with ten species ($S = 10$) and ten resources ($d = 10$). We started from a community where species were completely different in terms of resource utilization (**C** is an identity matrix), species obtained identical values from all resources (i.e., all $w_{ik} = 0.5$), and all resource dynamics parameters were identical (i.e., all $\theta_k = 0.5$). **a**, We then systematically increased similarity across species in a stepwise manner as depicted from left to right in the similarity tree. We first made the second species identical to the first species, and they showed complete niche overlap at the point indicated by the red circle. We next made the third species identical the first two species, and it become exactly identical at the blue square. Then the fourth species was merged into this group at the point indicated by the white diamond, and so on. **b**, At each step of this process, we used our SVD-based framework to estimate the inferred dimensionality $\hat{d}$ of the resulting interaction matrix. As expected, dimensionality at the start was equal to the initial number of limiting resources ($\hat{d} = 10$) and decreased as species showed increasing niche overlap. **c** & **d**, We show the underlying resource utilization matrices **C** and emergent interaction matrices **A** at the points x, y, and z indicated with the vertical dotted lines in panel **b**. In these sets of matrices, darker red colors indicate high resource utilization or strong interactions and lighter colors indicate low resource utilization or weaker interactions, respectively.

Supplementary Note 3

Additive representation of response–effect parameterized interactions

As described in the main text, our d-dimensional approximation of the $S \times S$ interaction matrix $\mathbf{A}$ is given by $\mathbf{A}_d = \mathbf{R}_d \, \boldsymbol{\Sigma}_d \, \mathbf{E}_d^T$. We will show here why this matrix representation is mathematically equivalent to Eq. (1) from the main text which states that the matrix $\mathbf{A}_d$ is composed of elements whose values are given by $\alpha_{ij|d} = \sum_{k=1}^{d} \sigma_k \, r_{i,k} \, e_{j,k}$. As a representative example, consider an assemblage of $S = 4$ species whose interactions are constrained to occur in $d = 2$ niche dimensions. The necessary parameters for this scenario include

$$\mathbf{R}_2 = \begin{bmatrix} r_{1,1} & r_{1,2} \\ r_{2,1} & r_{2,2} \\ r_{3,1} & r_{3,2} \\ r_{4,1} & r_{4,2} \end{bmatrix} ,$$

$$\mathbf{E}_2 = \begin{bmatrix} e_{1,1} & e_{1,2} \\ e_{2,1} & e_{2,2} \\ e_{3,1} & e_{3,2} \\ e_{4,1} & e_{4,2} \end{bmatrix} ,$$

and

$$\boldsymbol{\Sigma}_2 = \begin{bmatrix} \sigma_1 & 0 \\ 0 & \sigma_2 \end{bmatrix} .$$

The matrix $\mathbf{A}_2$ is obtained by multiplying these three matrices together as

$$\mathbf{A}_2 = \overbrace{\begin{bmatrix} r_{1,1} & r_{1,2} \\ r_{2,1} & r_{2,2} \\ r_{3,1} & r_{3,2} \\ r_{4,1} & r_{4,2} \end{bmatrix}}^{\mathbf{R}_2} \overbrace{\begin{bmatrix} \sigma_1 & 0 \\ 0 & \sigma_2 \end{bmatrix}}^{\boldsymbol{\Sigma}_2} \overbrace{\begin{bmatrix} e_{1,1} & e_{2,1} & e_{3,1} & e_{4,1} \\ e_{1,2} & e_{2,2} & e_{3,2} & e_{4,2} \end{bmatrix}}^{\mathbf{E}_2^T} ,$$

which is equal to

$$\mathbf{A}_2 = \begin{bmatrix} \sigma_1 r_{1,1} e_{1,1} + \sigma_2 r_{1,2} e_{1,2} & \sigma_1 r_{1,1} e_{2,1} + \sigma_2 r_{1,2} e_{2,2} & \sigma_1 r_{1,1} e_{3,1} + \sigma_2 r_{1,2} e_{3,2} & \sigma_1 r_{1,1} e_{4,1} + \sigma_2 r_{1,2} e_{4,2} \\ \sigma_1 r_{2,1} e_{1,1} + \sigma_2 r_{2,2} e_{1,2} & \sigma_1 r_{2,1} e_{2,1} + \sigma_2 r_{2,2} e_{2,2} & \sigma_1 r_{2,1} e_{3,1} + \sigma_2 r_{2,2} e_{3,2} & \sigma_1 r_{2,1} e_{4,1} + \sigma_2 r_{2,2} e_{4,2} \\ \sigma_1 r_{3,1} e_{1,1} + \sigma_2 r_{3,2} e_{1,2} & \sigma_1 r_{3,1} e_{2,1} + \sigma_2 r_{3,2} e_{2,2} & \sigma_1 r_{3,1} e_{3,1} + \sigma_2 r_{3,2} e_{3,2} & \sigma_1 r_{3,1} e_{4,1} + \sigma_2 r_{3,2} e_{4,2} \\ \sigma_1 r_{4,1} e_{1,1} + \sigma_2 r_{4,2} e_{1,2} & \sigma_1 r_{4,1} e_{2,1} + \sigma_2 r_{4,2} e_{2,2} & \sigma_1 r_{4,1} e_{3,1} + \sigma_2 r_{4,2} e_{3,2} & \sigma_1 r_{4,1} e_{4,1} + \sigma_2 r_{4,2} e_{4,2} \end{bmatrix} .$$

Note that each individual element in $\mathbf{A}_2$ is a sum across the two underlying niche dimensions. As a consequence, we could instead rewrite this matrix product as a sum of two matrices

$$\mathbf{A}_2 = \begin{bmatrix} \sigma_1 r_{1,1} e_{1,1} & \sigma_1 r_{1,1} e_{2,1} & \sigma_1 r_{1,1} e_{3,1} & \sigma_1 r_{1,1} e_{4,1} \\ \sigma_1 r_{2,1} e_{1,1} & \sigma_1 r_{2,1} e_{2,1} & \sigma_1 r_{2,1} e_{3,1} & \sigma_1 r_{2,1} e_{4,1} \\ \sigma_1 r_{3,1} e_{1,1} & \sigma_1 r_{3,1} e_{2,1} & \sigma_1 r_{3,1} e_{3,1} & \sigma_1 r_{3,1} e_{4,1} \\ \sigma_1 r_{4,1} e_{1,1} & \sigma_1 r_{4,1} e_{2,1} & \sigma_1 r_{4,1} e_{3,1} & \sigma_1 r_{4,1} e_{4,1} \end{bmatrix} + \begin{bmatrix} \sigma_2 r_{1,2} e_{1,2} & \sigma_2 r_{1,2} e_{2,2} & \sigma_2 r_{1,2} e_{3,2} & \sigma_2 r_{1,2} e_{4,2} \\ \sigma_2 r_{2,2} e_{1,2} & \sigma_2 r_{2,2} e_{2,2} & \sigma_2 r_{2,2} e_{3,2} & \sigma_2 r_{2,2} e_{4,2} \\ \sigma_2 r_{3,2} e_{1,2} & \sigma_2 r_{3,2} e_{2,2} & \sigma_2 r_{3,2} e_{3,2} & \sigma_2 r_{3,2} e_{4,2} \\ \sigma_2 r_{4,2} e_{1,2} & \sigma_2 r_{4,2} e_{2,2} & \sigma_2 r_{4,2} e_{3,2} & \sigma_2 r_{4,2} e_{4,2} \end{bmatrix}.$$

This sum can then be written even more compactly as

$$\mathbf{A}_2 = \sigma_1 \boldsymbol{r}_{\cdot,1} \boldsymbol{e}_{\cdot,1}^T + \sigma_2 \boldsymbol{r}_{\cdot,2} \boldsymbol{e}_{\cdot,2}^T ,$$

where $\boldsymbol{r}_{\cdot,k}$ is the k-th column of $\mathbf{R}_2$ and $\boldsymbol{e}_{\cdot,k}$ is the k-th column of $\mathbf{E}_2$.

Returning to the general case, this exercise implies that the matrix product $\mathbf{A}_d = \mathbf{R}_d \, \boldsymbol{\Sigma}_d \, \mathbf{E}_d^T$ is mathematically equivalent to $\mathbf{A}_d = \sum_{k=1}^{d} \sigma_k \, \boldsymbol{r}_{\cdot,k} \, \boldsymbol{e}_{\cdot,k}^T$, where the sum is across "interaction matrices" at each niche dimension. On an element-wise basis, this is identical to our assertion that $\alpha_{ij|d} = \sum_{k=1}^{d} \sigma_k \, r_{i,k} \, e_{j,k}$.

Supplementary Note 4

Niche dimensionality of empirical datasets

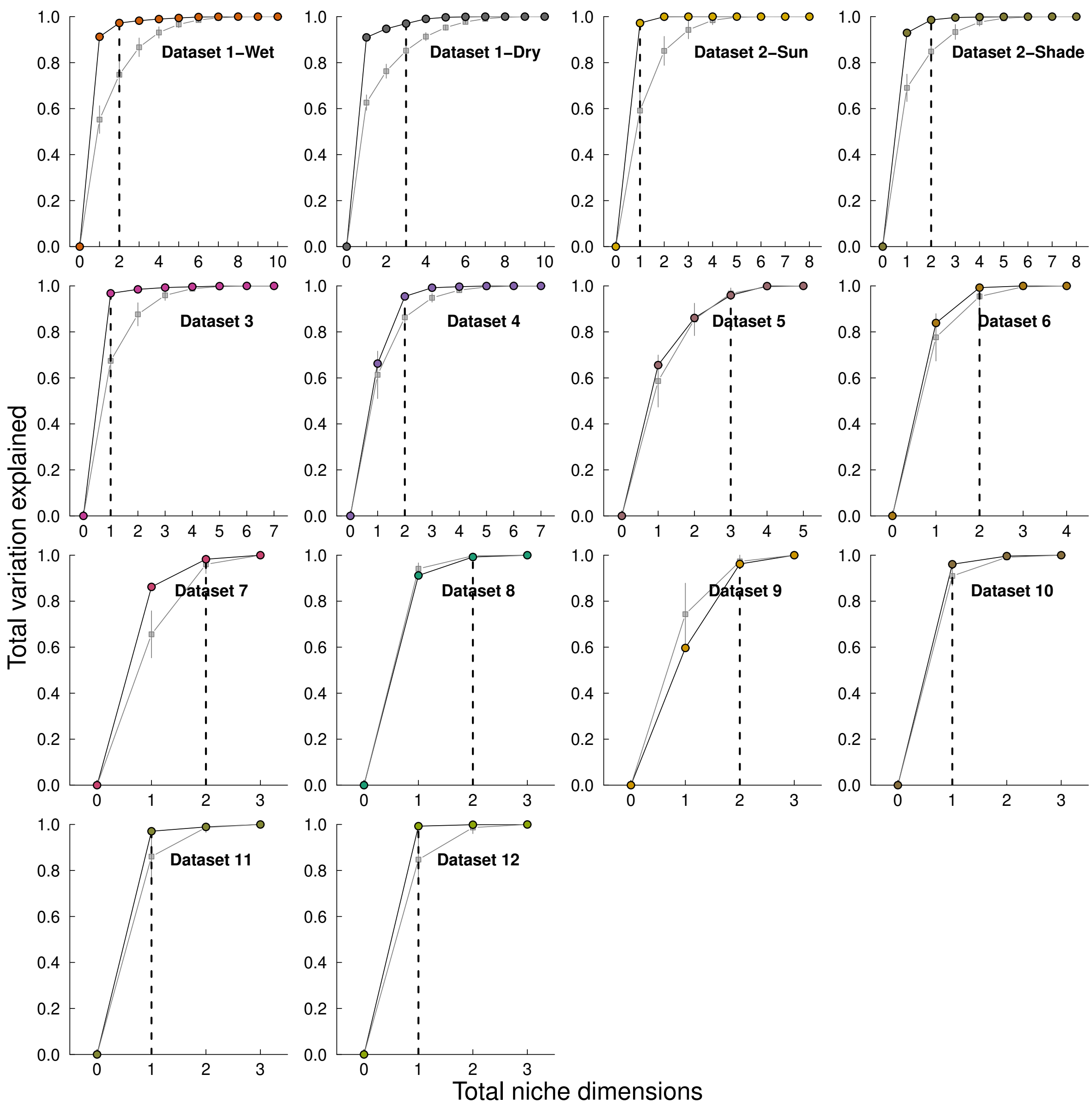

Figure S9: For each dataset, we show the total variance explained when interactions are inferred at a fixed number of niche dimensions (colored circles and black lines). The dashed line indicates the inferred niche dimensionality $\hat{d}$ required to explain at least 95% of the variation in the observed interactions. The grey circles and grey lines provide the same information for randomizations of the observed interaction matrices (mean ± two standard deviations).

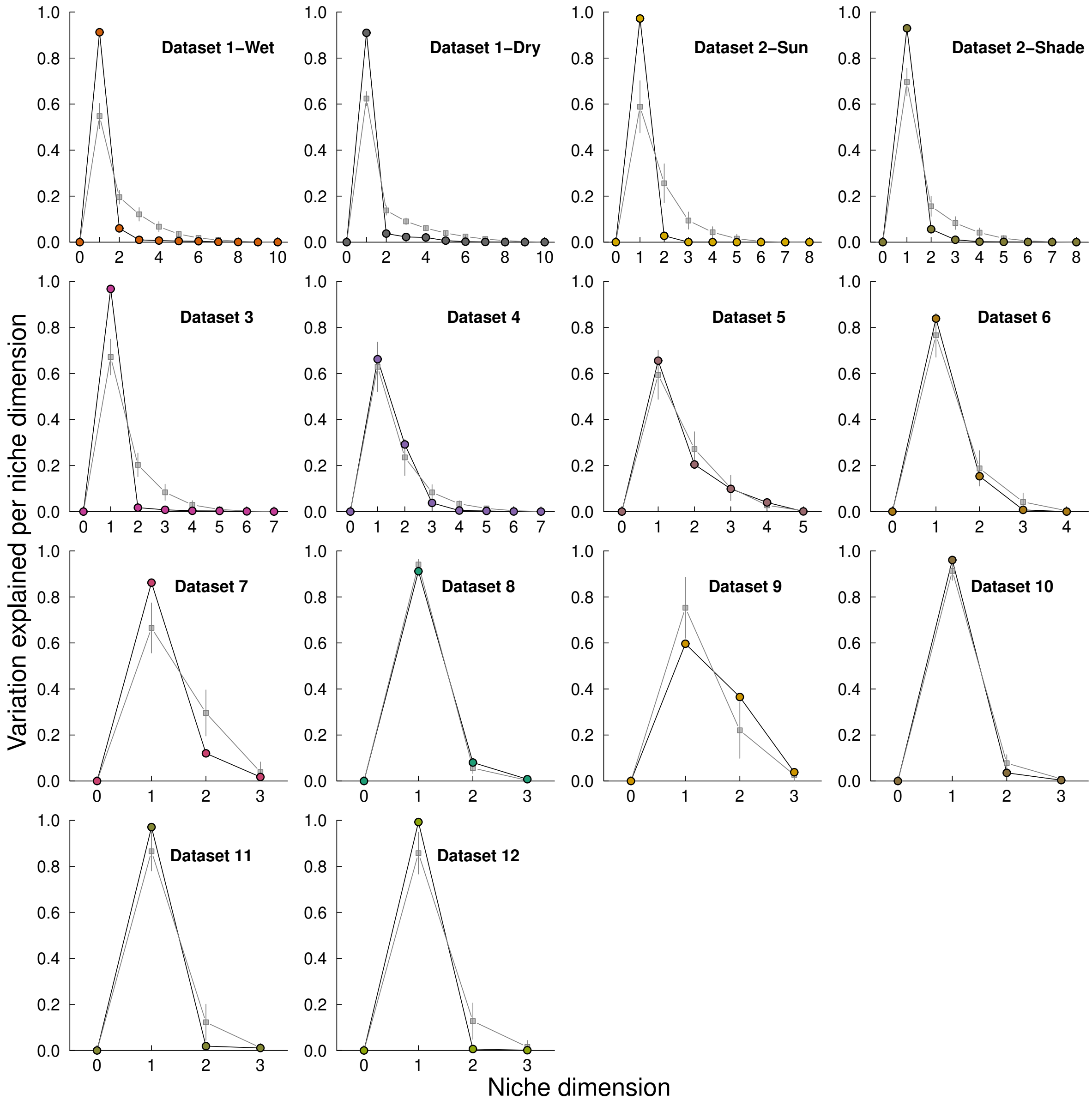

Figure S10: For each dataset, we show the incremental variance explained by each niche dimension when interactions are inferred at niche dimensionality $d = S$ (colored circles and black lines). The grey circles and grey lines provide the same information for randomizations of the observed interaction matrices (mean $\pm$ two standard deviations).

Table S1: Ordered list of singular values for each of the empirical datasets studied here.

Dataset	σ_1	σ_2	σ_3	σ_4	σ_5	σ_6	σ_7	σ_8	σ_9	σ_{10}
1-Wet	25.56	6.554	2.682	2.233	1.857	1.715	0.9239	0.6258	0.05918	5.353e-18
1-Dry	14.81	3.001	2.335	2.221	1.278	0.71	0.475	0.1039	0.02807	1.192e-17
2-Sun	193.4	32.58	3.576	2.04	0.1918	0.06573	0.02544	3.896e-06	NA	NA
2-Shade	39.4	9.686	4.18	1.835	1.45	0.8753	0.4685	0.0002487	NA	NA
3	19.81	2.642	1.774	1.178	1.084	0.5971	0.1022	NA	NA	NA
4	24.81	16.47	5.924	2.06	1.653	0.6375	0.1506	NA	NA	NA
5	1.1	0.615	0.4272	0.271	0.03756	NA	NA	NA	NA	NA
6	3.885	1.663	0.3665	0.01924	NA	NA	NA	NA	NA	NA
7	2.127	0.7947	0.3035	NA	NA	NA	NA	NA	NA	NA
8	1.489	1.165	0.377	NA	NA	NA	NA	NA	NA	NA
9	4.893	1.453	0.4545	NA	NA	NA	NA	NA	NA	NA
10	2.051	0.2853	0.2148	NA	NA	NA	NA	NA	NA	NA
11	4.57	0.8801	0.2893	NA	NA	NA	NA	NA	NA	NA
12	9.367	0.7454	0.303	NA	NA	NA	NA	NA	NA	NA

Supplementary Note 5

Observed versus fit interactions for all datasets

Figure S11: Observed and fit interactions for each empirical dataset. On the left of each row, we show the observed interaction matrix. To the right of the observed matrix, we show the inferred interaction matrix at niche dimensionality $\hat{d}$ as noted in Table 1. Interaction strengths have been scaled by dataset such that the most competitive is red, the most facilitative is blue, and no pairwise effect is white as noted in the colorbars on the far right.

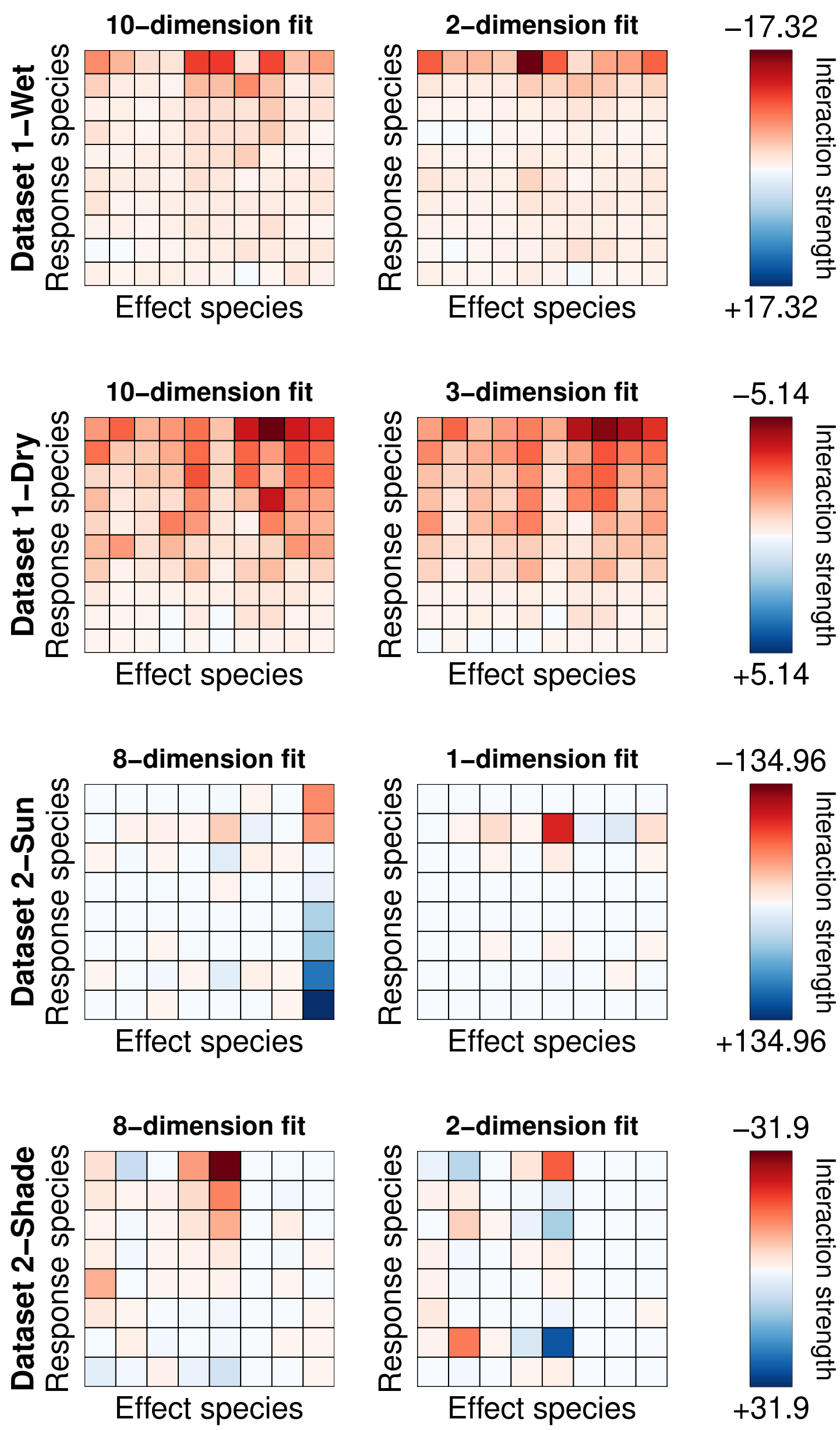

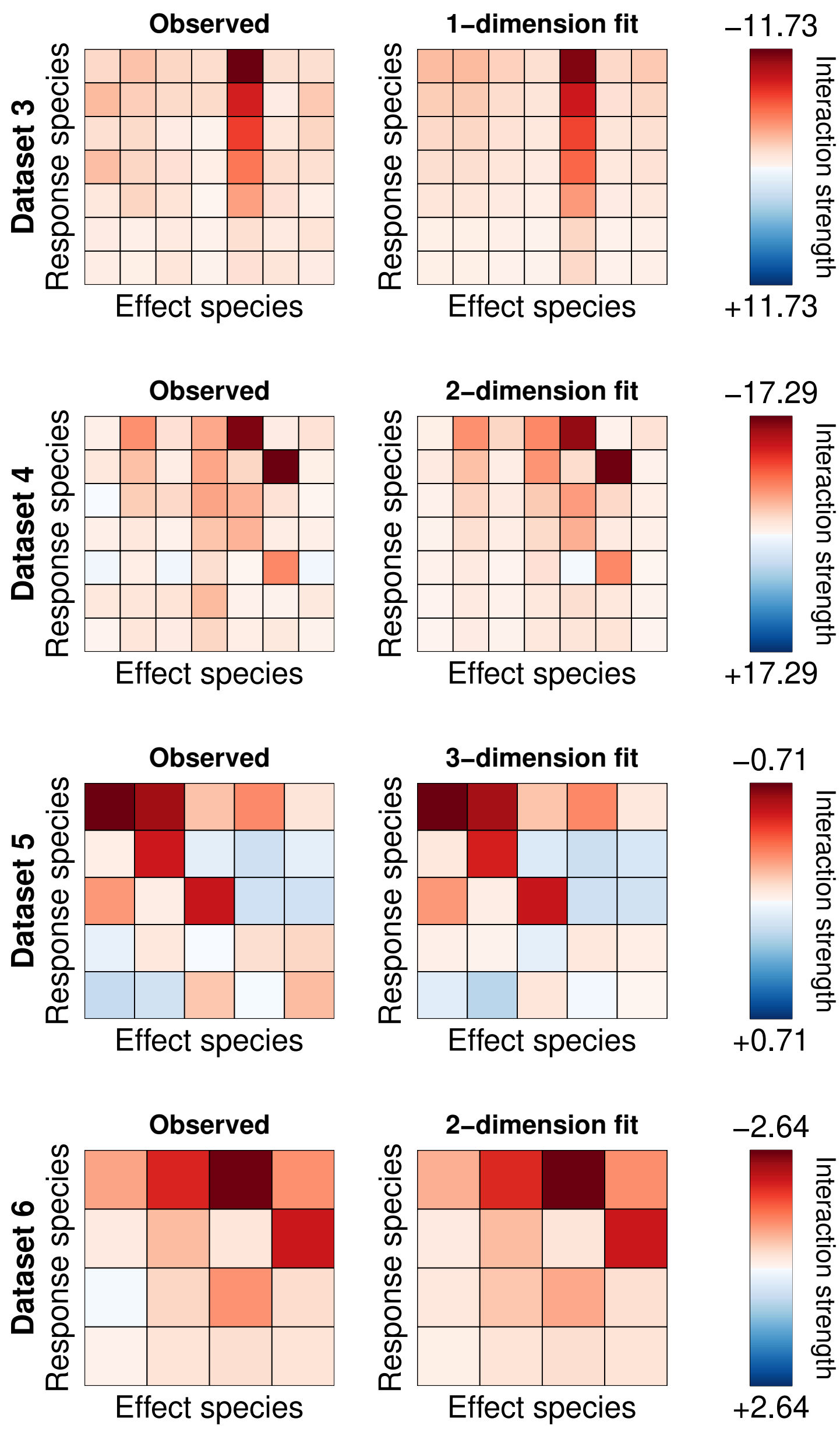
Dataset 3
Observed
1–dimension fit
Dataset 4
Observed
2–dimension fit
Dataset 5
Observed
3–dimension fit
Dataset 6
Observed
2–dimension fit
Response species
Effect species
Interaction strength
−11.73
+11.73
−17.29
+17.29
−0.71
+0.71
−2.64
+2.64

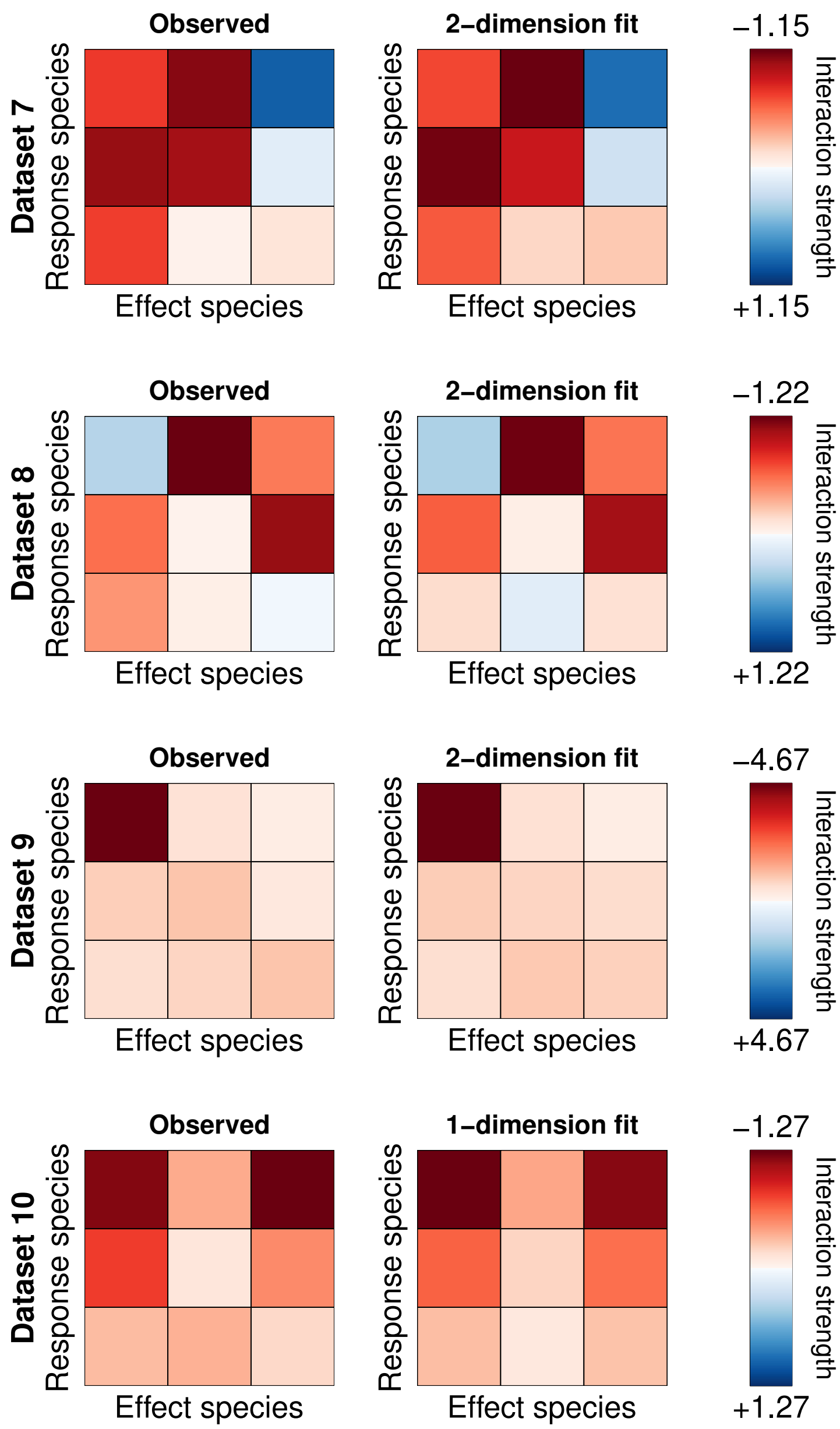
Dataset 7
Observed
2–dimension fit
Response species
Effect species
–1.15
+1.15
Interaction strength
Dataset 8
Observed
2–dimension fit
Response species
Effect species
–1.22
+1.22
Interaction strength
Dataset 9
Observed
2–dimension fit
Response species
Effect species
–4.67
+4.67
Interaction strength
Dataset 10
Observed
1–dimension fit
Response species
Effect species
–1.27
+1.27
Interaction strength

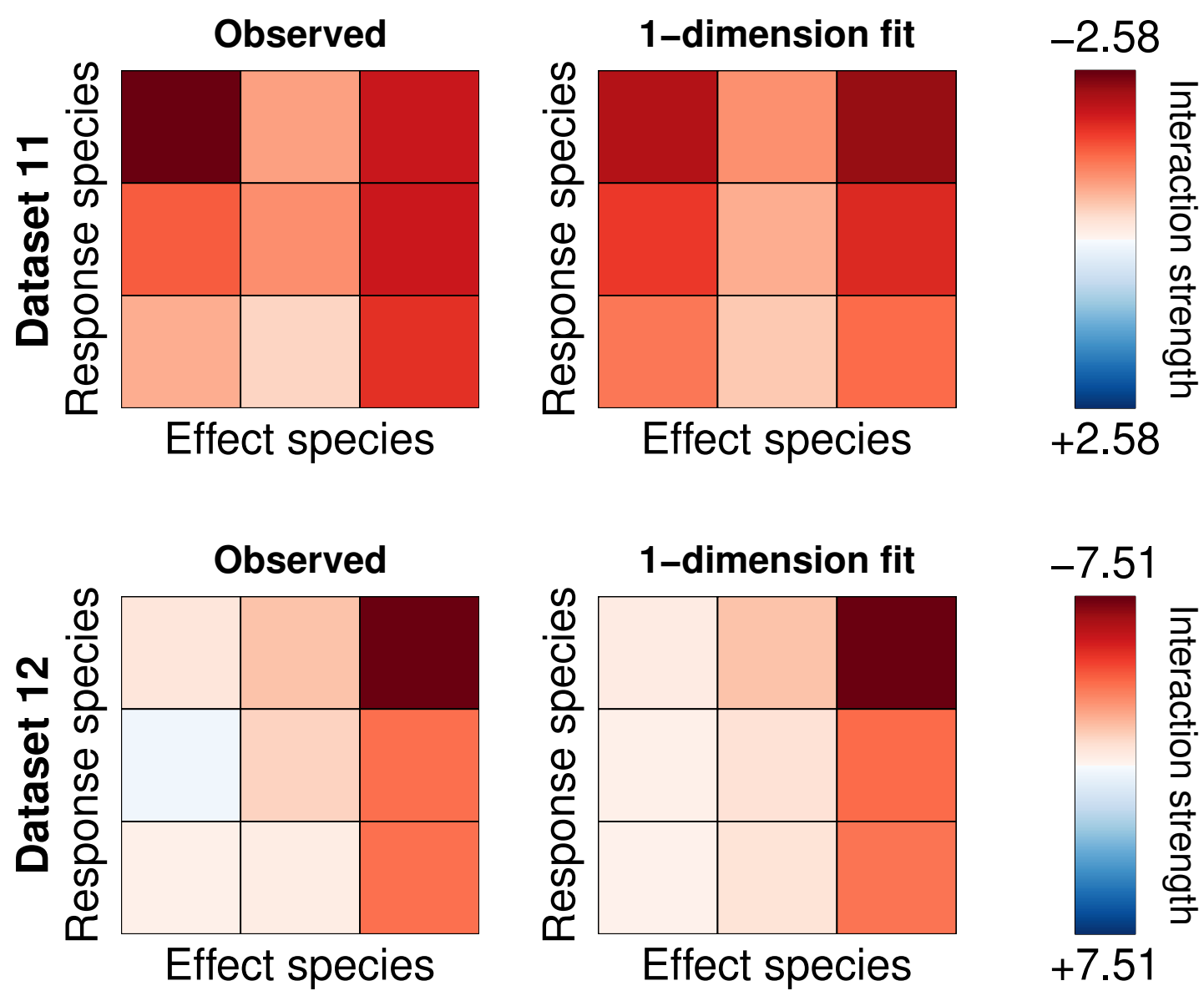
Observed
1−dimension fit
Dataset 11
Response species
Effect species
Response species
Effect species
−2.58
+2.58
Interaction strength
Observed
1−dimension fit
Dataset 12
Response species
Effect species
Response species
Effect species
−7.51
+7.51
Interaction strength

Supplementary Note 6

Niche dimensionality of randomised data

Just as random data may occasionally be perfectly correlated (or perfectly uncorrelated), we wished to ensure that our observation of low niche dimensionality ($\hat{d}$) across the various empirical assemblages was not just a spurious outcome of our methodology. To check this, we performed two separate tests.

First, we used Dataset 3 as a template from which to generate an ensemble of 25 randomized "equivalents" for which we could estimate $\hat{d}$. This dataset had 7 focal species, and we sampled their intrinsic growth rates λ_i from a uniform distribution $U(0, 10)$ and their pairwise interaction coefficients α_{ij} from a uniform distribution $U(0, 1)$. We then simulated biomass production using Eq. (1) for each focal species growing with neighbour densities $N_j \in 1, 5, 10, 25, 50, 75, 100, 175, 250$—which is equivalent to the experimental conditions for the observed data[10]. We simulated one replicate observation for each focal–neighbour combination, giving a total of 441 simulated observations per randomized dataset. For each of these, we then estimated $\hat{d}$ based on the fit with lowest AIC. Our expectation was that $\hat{d}$ should tend to be equal to S since the randomized data lacks a consistent underlying structure and/or competitive hierarchy. As expected, we found that the median $\hat{d} = 7$ (mode = 7; mean = 6.36; variance = 0.74) across these 25 randomized datasets (Fig. S12).

Since the process above creates highly unstructured data, it likely provides an overly conservative test of our methodology. As a second test, we therefore also took the empirical interaction matrices, generated an ensemble of 250 randomizations for each by randomly shuffling the placement of the values in each matrix, and the followed the steps described in the Methods to identify the niche dimensionality of each of these randomized

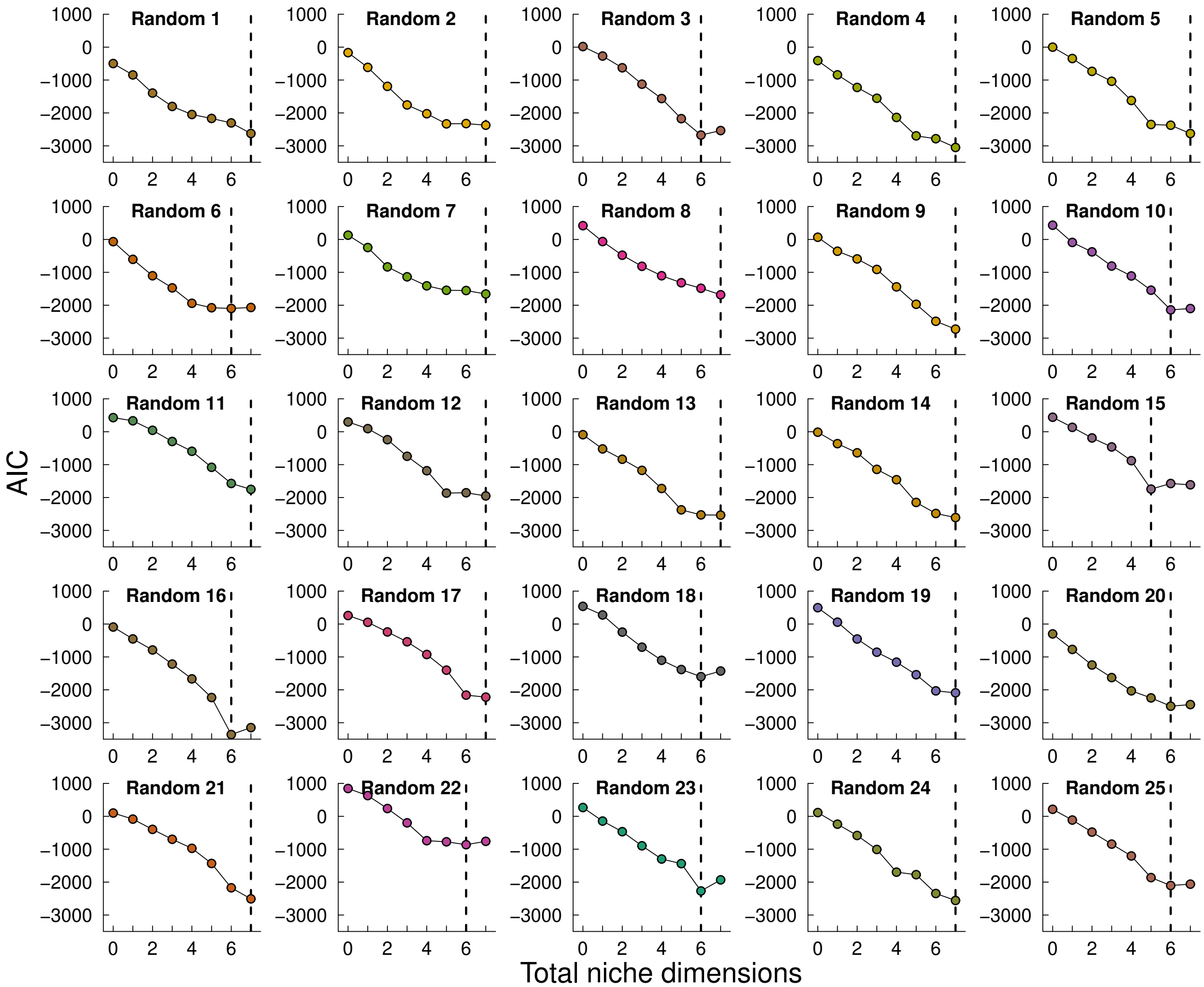

Figure S12: For an ensemble of randomly-generated data, we show the AIC of model fits at fixed numbers of niche dimensions. The dashed line indicates the niche dimensionality $\hat{d}$ corresponding to minimum AIC.

matrices. For a majority of empirical datasets, the randomizations were again more "unstructured" than were the empirical interaction matrices such that a greater number of niche dimensions were necessary to capture 95% of the variation within the randomized data. Across all datasets, this led to consistent trend of greater inferred niche dimensionality for randomized data than empirical data when interaction matrices had the same

number of species (Fig. S13).

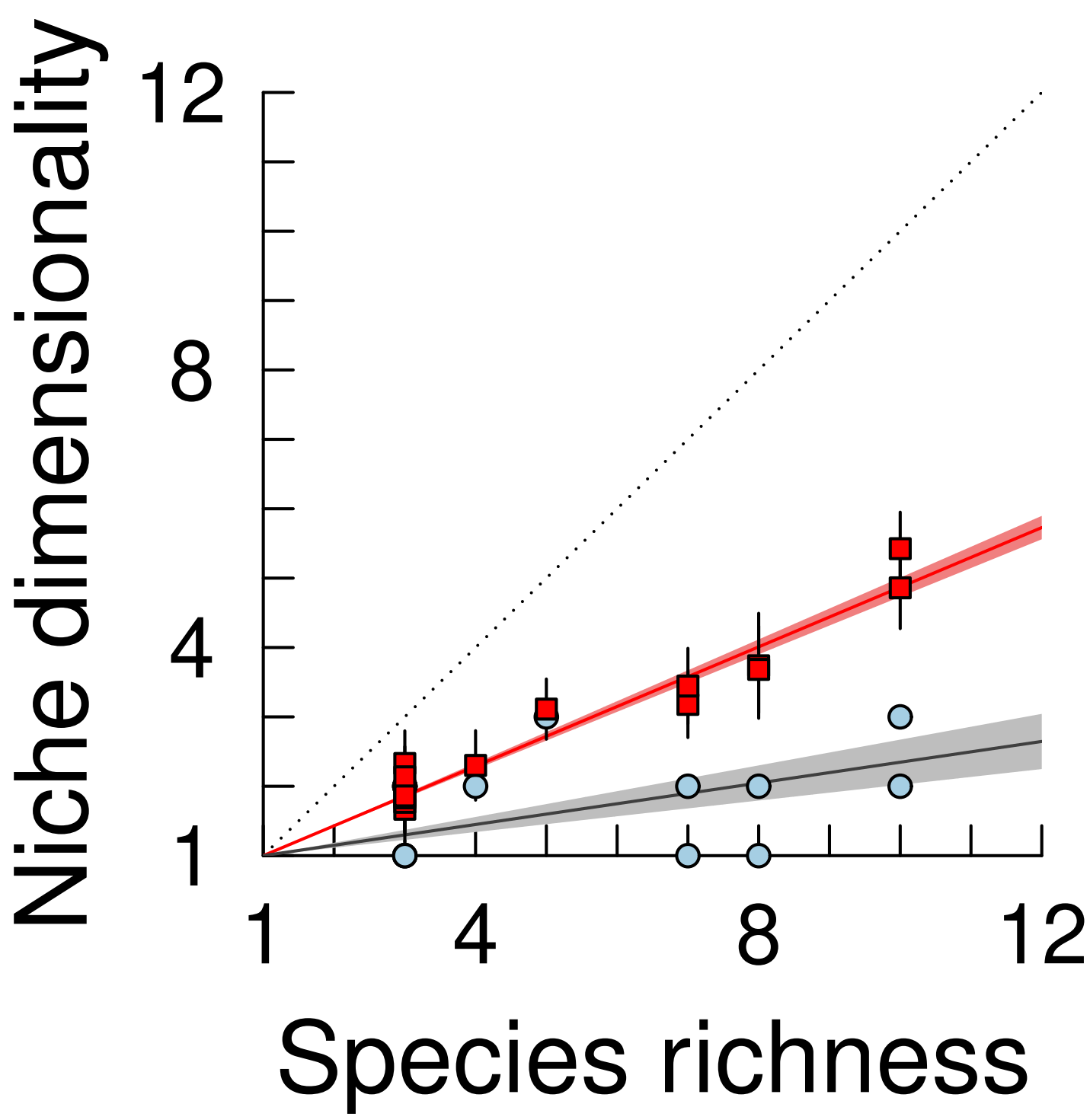

Figure S13: Inferred niche dimensionality ($\hat{d}$) relative to the actual species richness of each empirical assemblage (S), for both the empirical data and randomizations thereof. As in Fig. 3 from the main text, the dotted line represents the upper bound of niche dimensionality equal to the number of species. The solid black line (grey shaded region) represents the predicted increase of niche dimensions ($\pm$ standard error) based on the trend observed across the empirical datasets (blue dots). The solid red line (red shaded region) represents the predicted increase of niche dimensions ($\pm$ standard error) based on the trend observed across the randomized datasets (red dots; mean $\pm$ standard error). Note that both trend lines have been constrained to pass through $\hat{d} = 1$ when $S = 1$ since a one species assemblage necessarily requires only a single niche dimension.

Supplementary Note 7

Response and effect traits for all datasets

Figure S14: The relative position of all species in each dataset in response–effect trait space. Each row corresponds to a different dataset and is formatted as in Fig. 2 for the first trait dimension of Dataset 1-Wet and Dataset 1-Dry. Each point corresponds to the mean value for a species and error bars extend from the 25th percentile to the 75th percentile. Recall that the number of response and effect traits for each species is equivalent to the inferred niche dimensionality $\hat{d}$ of the assemblage (Table 1). Therefore, some datasets (rows) necessarily have fewer panels than others. Likewise, matrix-only datasets lead to deterministic response and effect traits and hence have no associated error bars.

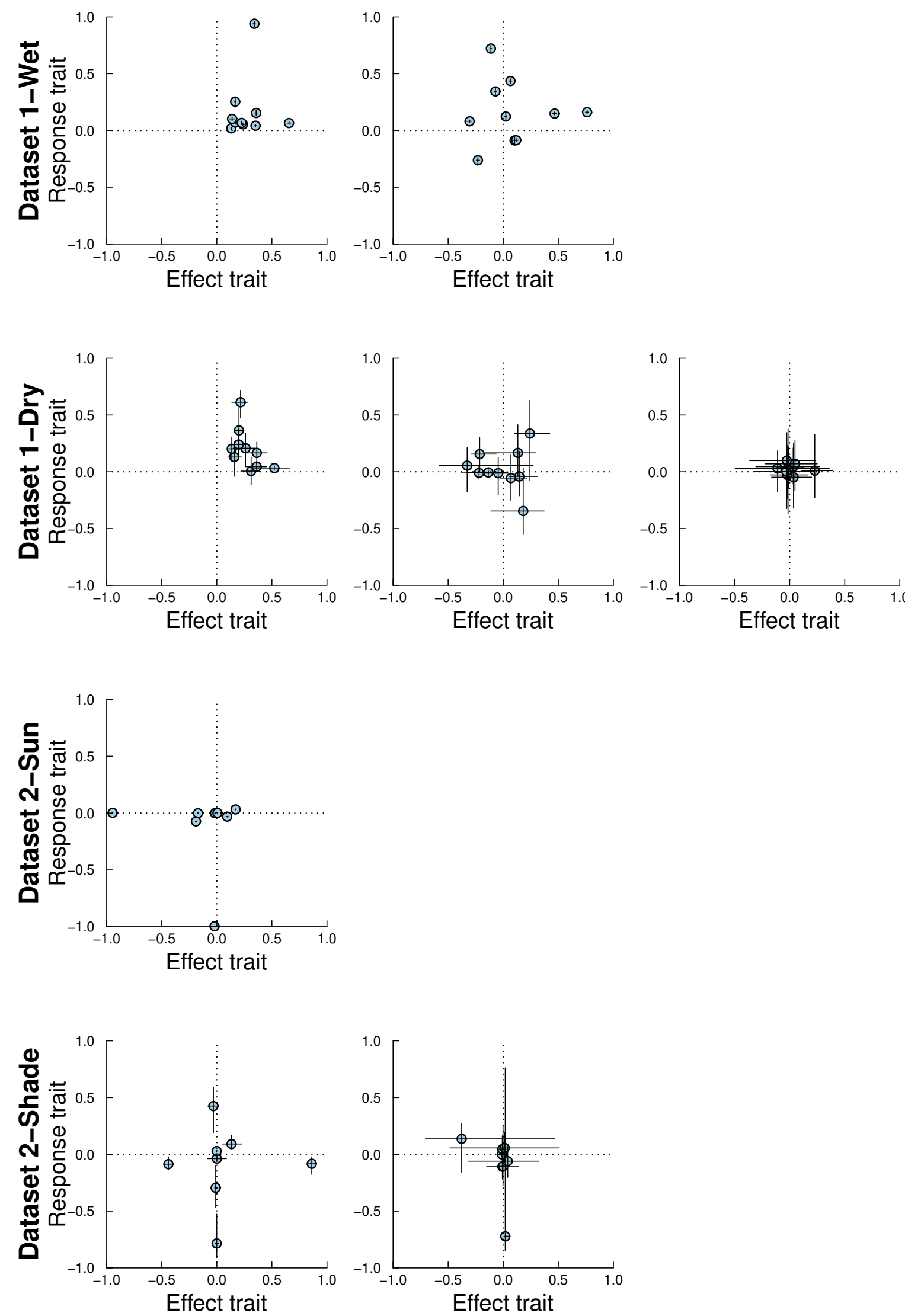

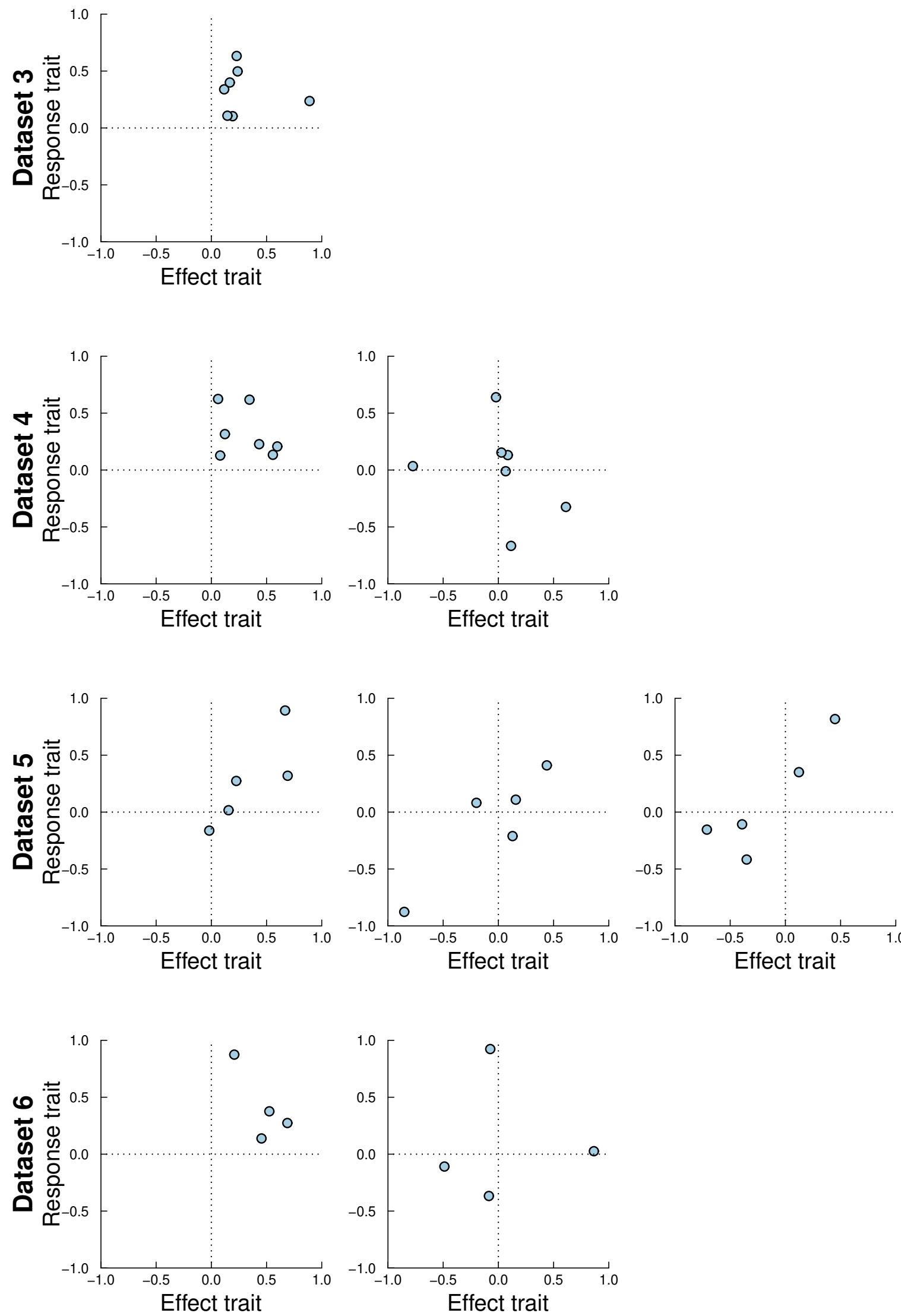
Dataset 3
Dataset 4
Dataset 5
Dataset 6
Response trait
Effect trait

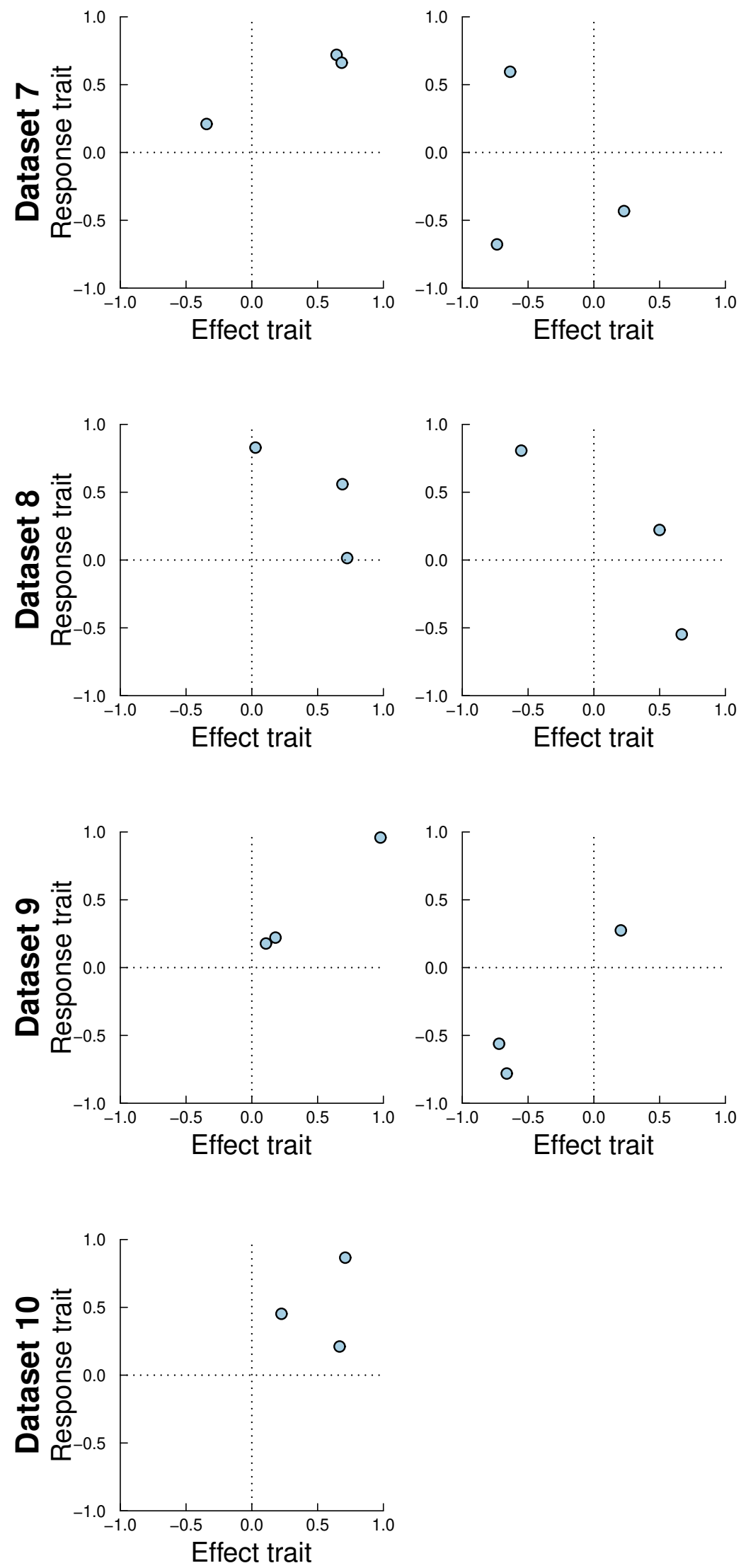
Dataset 7
Dataset 8
Dataset 9
Dataset 10
Response trait
Effect trait
1.0
0.5
0.0
−0.5
−1.0

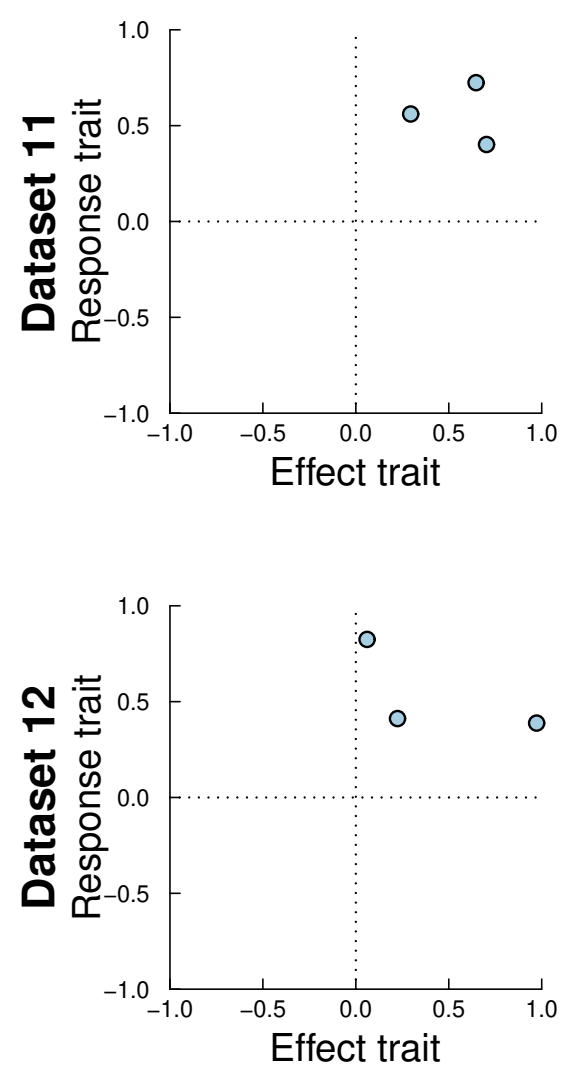
Dataset 11
Response trait
Effect trait
1.0
0.5
0.0
−0.5
−1.0
Dataset 12
Response trait
Effect trait

Supplementary Note 8

Simulation model

To explore the consequences that environmentally driven variation in species' response andeffect traits might have for coexistence outcomes, we combined our reduced-dimensionality parameterization of the interaction matrix $\mathbf{A}$ with a well-known model for annual-plant population dynamics[11,12]. In this model, the year-to-year population dynamics of viable seeds in the seed bank for each species is given by

$$\frac{N_{i,t+1}}{N_{i,t}} = (1-g_i)s_i + \frac{g_i \lambda_i}{1+\sum_j \alpha_{ij} g_j N_{j,t}}\,, \tag{S6}$$

where $N_{i,t}$ is the density of seeds from species i in year t, g_i is the annual germination rate of seeds from species i, s_i is the annual survival probability of ungerminated seeds from species i, λ_i is the per capita seed production of plants from species i in the absence of competition, and α_{ij} is the per capita effect of plants from species j on the fecundity of plants from species i. Note that both λ_{ij} and α_{ij} are defined as in Eq. (1) from the main text.

For simplicity, we consider an assemblage consisting of just two species and that experiences two distinct types of environments (Dry and Wet). We parameterized the model so that in both environment types the two species have identical germination rates ($g_i = g_j = 0.5$), identical seed survival rates ($s_i = s_j = 0.8$), and identical intrinsic fecundities ($\lambda_i = \lambda_j = 15$). We also constrained the interactions to occur in a single niche dimension with strength $\sigma_1 = 1$. In both environment types, the two species have equivalent effect traits $e_{i,1} = e_{j,1}$. In the Dry environment, the response trait of species i is twice that of species j (i.e., $r_{i,1}^{\mathrm{Dry}} = 2\,r_{j,1}^{\mathrm{Dry}}$) implying that species i experiences stronger competitive effects in the Dry environment; in the Wet environment, the response trait of species j is twice that of species i (i.e., $r_{j,1}^{\mathrm{Wet}} = 2\,r_{i,1}^{\mathrm{Wet}}$) implying that species j experiences stronger

competitive effects in the Wet environment. Note that the orthogonality constraints from our parameterization require that $r_{i,1}^{\text{Dry}} = r_{j,1}^{\text{Wet}} = 1/\sqrt{5}$ and $e_{i,1} = e_{j,1} = 1/\sqrt{2}$.

Given this parameterization, species j is predicted to dominate in Dry environments and species i is predicted to dominate in Wet environments (Fig. S15). This is because, with a $d = 1$ dimension interaction matrix, the two species exhibit no niche differentiation; however species j has a fitness advantage in the Dry environment and species i has a fitness advantage in the Wet environment[13]. In constant Dry or Wet environments, the dominant species will therefore eventually competitively exclude the other over time as long if the model starts with a feasible initial condition (i.e., $N_{i,0} > 0$ and $N_{j,0} > 0$). If the environment experiences year-to-year variation, however, it becomes possible for both species to persist in the long run (Fig. S15). In the ecological literature, this is called a "storage effect"[14] and is used to refer to the idea that species-specific responses to varying environmental conditions can allow them to buffer against unfavorable environments. These simulations thus provide an example of a "temporal" storage effect since environmental variation changes across time[15]; however, a similar phenomena can occur over space, for example if the species interact in an landscape comprising Dry and Wet patches[16].

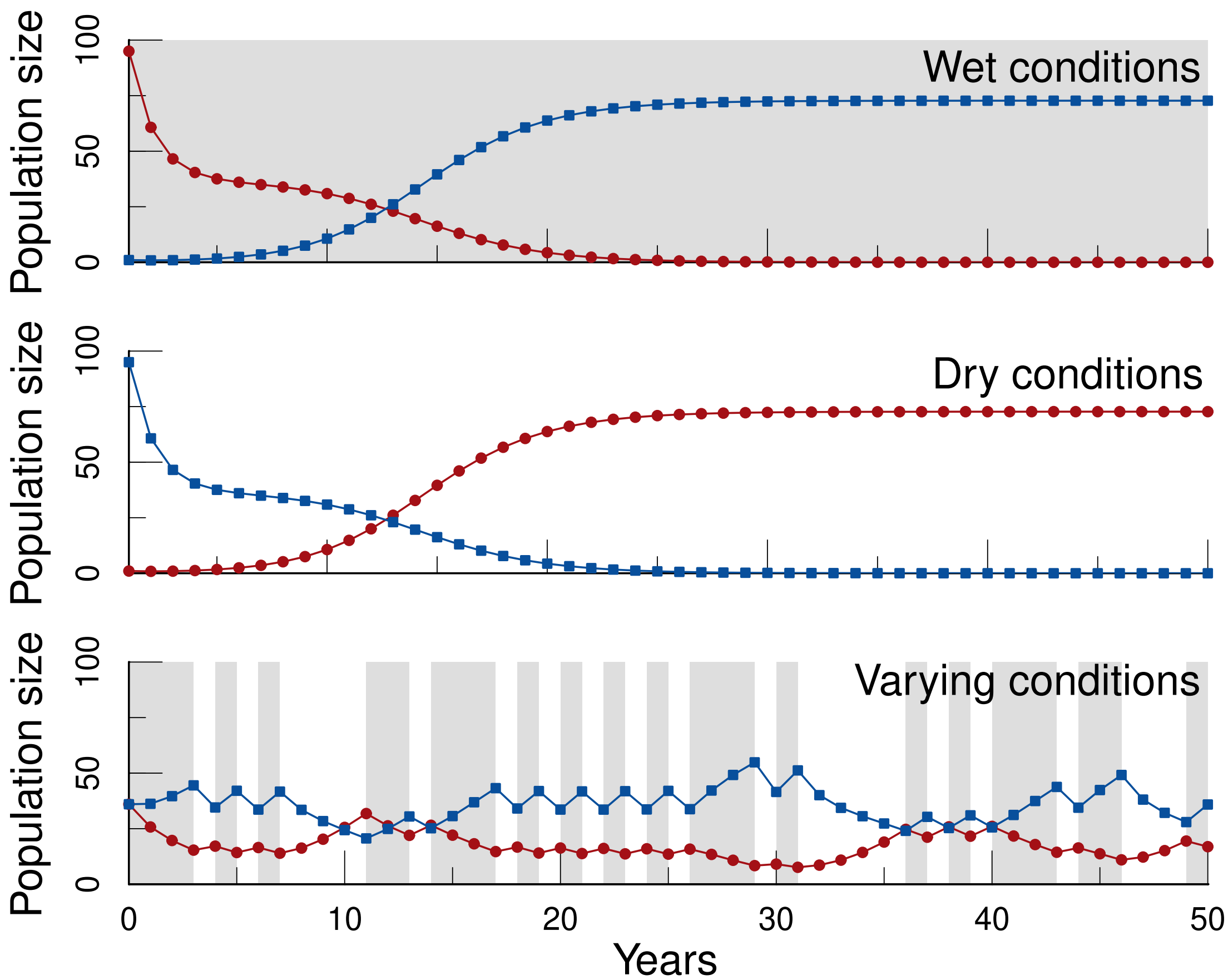

Figure S15: Species coexistence with low dimensional interactions can be facilitated by varying environmental conditions. **Top**, Under consistently Wet conditions, species j (red circles) is outcompeted by species i (blue squares) because its higher response trait creates a fitness disadvantage, even if species j is initially more abundant ($N_{i,0} = 1$ and $N_{j,0} = 95$). **Middle**, Under consistently Dry conditions, species j outcompetes species i because its lower response trait creates a fitness advantage, even if species i is initially more abundant ($N_{i,0} = 95$ and $N_{j,0} = 1$). **Bottom**, If conditions fluctuate randomly between Dry and Wet conditions, it is possible for species i and species j to overcome lack of niche differentiation and maintain positive population sizes over time. Though such fluctuations cannot guarantee coexistence over an infinite time frame, they can substantially decrease the probability of competitive exclusion. In all panels, the grey shading indicates Wet years whereas the white background indicates Dry years.

Supplementary References

1. Connell, J. H. Apparent versus "real" competition in plants. In *Perspectives in Plant Competition* (eds. Grace, J. & Tilman, D.), 9–26 (Academic Press, Inc., San Diego, CA, USA, 1990).

2. Goldberg, D. E. Components of resource competition in plant communities. In *Perspectives in Plant Competition* (eds. Grace, J. & Tilman, D.), 27–49 (Academic Press, Inc., San Diego, CA, USA, 1990).

3. Adler, P. B. *et al.* Competition and coexistence in plant communities: intraspecific competition is stronger than interspecific competition. *Ecol. Lett.* **21**, 1319–1329 (2018).

4. MacArthur, R. Species packing and competitive equilibrium for many species. *Theor. Popul. Biol.* **1**, 1–11 (1970).

5. Chesson, P. MacArthur's consumer-resource model. *Theor. Popul. Biol.* **37**, 26–38 (1990).

6. Cheng, H., Gimbutas, Z., Martinsson, P. G. & Rokhlin, V. On the compression of low rank matrices. *SIAM J. Sci. Comput.* **26**, 1389–1404 (2005).

7. Legendre, P. & Legendre, L. *Numerical Ecology* (Elsevier, Amsterdam, The Netherlands, 2012).

8. McPeek, M. A. Mechanisms influencing the coexistence of multiple consumers and multiple resources: resource and apparent competition. *Ecol. Monogr.* **89**, e01328 (2019).

9. Barabás, G., Pásztor, L., Meszéna, G. & Ostling, A. Sensitivity analysis of coexistence in ecological communities: theory and application. *Ecol. Lett.* **17**, 1479–1494 (2014).

10. Goldberg, D. E. & Landa, K. Competitive effect and response: Hierarchies and correlated traits in the early stages of competition. *J. Ecol.* **79**, 1013–1030 (1991).

11. Cohen, D. Optimizing reproduction in a randomly varying environment. *J. Theor. Biol.* **12**, 119–129 (1966).

12. Watkinson, A. R. Density-dependence in single-species populations of plants. *J. Theor. Biol.* **83**, 345–357 (1980).

13. Godoy, O. & Levine, J. M. Phenology effects on invasion success: insights from coupling field experiments to coexistence theory. *Ecology* **95**, 726–736 (2014).

14. Chesson, P. L. Coexistence of Competitors in a Stochastic Environment: The Storage Effect. In *Population Biology* (eds. Freedman, H. I. & Strobeck, C.), 188–198 (Springer-Verlag, Berlin, Germany, 1983).

15. Letten, A. D., Dhami, M. K., Ke, P.-J. & Fukami, T. Species coexistence through simultaneous fluctuation-dependent mechanisms. *Proc. Natl. Acad. Sci. USA* **115**, 6745–6750 (2018).

16. Sears, A. L. W. & Chesson, P. New methods for quantifying the spatial storage effect: An illustration with desert annuals. *Ecology* **88**, 2240–2247 (2007).
